# Antimicrobial and antiproliferative activity of biosynthesized manganese nanocomposite with natural derivative originated by endophytic *Aspergillus terreus*

**DOI:** 10.1186/s12934-025-02651-x

**Published:** 2025-02-04

**Authors:** Nashwa El-Gazzar, Reem Farouk, Nervana S. Diab, Gamal Rabie, Basel Sitohy

**Affiliations:** 1 Department of Botany and Microbiology, Faculty of Science, Zagazig University, Zagazig, 44519 Egypt; 2 Department of Biochemistry, Children Hospital, Faculty of Medicine, Mansoura University, Mansoura, 35516 Egypt; 3 Department of Clinical Microbiology, Infection and Immunology, Umeå University, 90185 Umeå, Sweden; 4 Department of Diagnostics and Intervention, Oncology, Umeå University, 90185 Umeå, Sweden

**Keywords:** *Aspergillus terreus*, Natural antimicrobial products, 3-(2-Hydroxy-4,4-dimethyl-6-oxo-1-cyclohexen-1-yl)-4-oxopentanoic acid, Manganese nanoparticles, Nanocomposites, Antimicrobial activity, Anticancer activity

## Abstract

**Background:**

Scientists have faced difficulties in synthesizing natural substances with potent biological activity from cost-effective sources. Endophytic fungi metabolites with nanoparticles have been utilized to develop a friendly, suitable procedure to address this problem and ameliorate the average amount of antioxidant, antimicrobial, and anticancer materials. Therefore, this study utilized endophytic fungi as a source of the natural extract with biosynthesized manganese nanoparticles (MnNPs) in the form of nanocomposites.

**Methods:**

Thirty endophytic fungi were isolated and were assessed for their antioxidant activity by 1, 1-Diphenyl-2-picrylhydrazyl (DPPH) and antimicrobial activity. The most potent isolate was identified utilizing 18S rRNA and was applied to purify and separate their natural antimicrobial products by Flash column chromatography. In addition, the most potent product was identified based on instrumental analysis through Nuclear magnetic resonance (NMR), Fourier-transform infrared (FTIR), and Gas chromatography-mass spectrometry (GC.MS). The purified product was combined with biosynthsesized manganese nanoparticles (MnNPs) for the production of nanocomposite (MnNCs). Later on, the physicochemical features of MnNPs and its MnNCs were examined and then they were assessed for determination their biological activities.

**Results:**

The most potent isolate was identified as *Aspergillus terreus* with accession number OR243300. The antioxidant and antimicrobial product produced by the strain *A. terreus* was identified as an natural derivative consisting of 3-(2-Hydroxy-4,4-dimethyl-6-oxo-1-cyclohexen-1-yl)-4-oxopentanoic acid (HDOCOX) with the chemical formula C_13_H_18_O_5_. Furthermore, purified HDOCOX, MnNPs and Mn-HDOCOX-NPs nanocomposite (MnNCs) showed significant antimicrobial effectiveness. The minimum inhibitory concentrations (MICs) determined for MnNCs were 10 µg/mL against *C. albicans* and *E.coli*. Furthermore, MnNCs were reduced hepatocellular carcinoma viability.

**Conclusion:**

The use of HDOCOX, either alone or in combination with MnNPs, is a potential candidate for inhibiting pathogenic microbes and the development of an anticancer drug pipeline.

**Supplementary Information:**

The online version contains supplementary material available at https://doi.org/10.1186/s12934-025-02651-x.

## Background

At present, the prolonged use and abuse of conventional antibiotics are exacerbating the global health threat of antimicrobial resistance (AMR) [1, 2]. In addition, cancer treatments like chemotherapy and radiotherapy have a number of drawbacks have many side effects, which results in prolonged threats of the patient’s life [3]. This elucidates the urgent need for further investigation of natural resources to develop novel and efficient antimicrobial and anticancer agents [4–7]. In this context, endophytic fungi are the natural resources of several bioactive compounds that can be examined for their antimicrobial and anticancer potential [8].

Endophytic fungi are known to colonize plant tissues without posing any health risks to the plant [9–11]. There is a growing interest in endophytic fungi due to their extensive variation and their capacity to introduce the most potent products among their secondary metabolites [12–14]. Certain endophytic fungi produce many therapeutically useful compounds, such as steroids, terpenoids, flavonoids, quinines, lignins, alkaloids, phenylpropanoids, phenolic acid, phenol, peptides, chlorinated metabolites, aliphatic substances. These compounds are comparable to those generated by host plants [10]. The majority of these chemicals have beneficial properties for pharmacological or therapeutic applications, including antimicrobials, anthelmintics, pesticides, antitumors, immunosuppressants, and antioxidants [15–17]. Endophytic fungi crude extracts from culture broths have shown a cytotoxic effect against human cell lines, as well as their antimicrobial properties towards pathogenic *P. aeruginosa*, *E. coli*, *S. aureus,* and *C. albicans* [13, 15].

Furthermore, nanomaterials have garnered significant interest in medicinal applications as antimicrobial and cytotoxic agents [18–20]. This is due to their rapid and efficient adsorption in biological systems compared to the larger macromolecules [21, 22]. Among nanoparticles, metal oxide nanoparticles, especially MnO, have gathered concern because of their electromagnetic characteristics, bioactivity, and reduced toxicity on normal cells. For instance, the MnNPs were found to exhibit potent antibacterial efficiency [23]. In addition, nanocomposites demonstrated promising results in inhibiting pathogenic bacteria [7]. The mixed combinations of nanoparticles with natural agents showed efficient antimicrobial and/or antiproliferative agents [7, 13, 24].

Based on the findings of previous studies, the main objective of this study is to isolate endophytic fungi from medicinal plants and the separation of the active compounds immediately in the secondary products of the most potent fungus. The present work primarily investigated the biosynthesis, characterization, and evaluation of the antimicrobial and antiproliferative activities of MnNPs and MnNCs (MnNPs and the active fungal compound).

## Materials and methods

### Samples collection and preparation

For endophytic fungi isolation, leaves of *Moringa oleifera* Lam., *Psidium guava* L.*, Medicago sativa* L.*, Beta vulgarus* L.*,* and *Ocimum basilicum* L., were collected and obtained from Plant Protection Research Institute, Agriculture Research Centre, Sharkia, Egypt, for the study during May/2020. The plants were identified by. Dr. Samir S. Teleb, Assistant Professor of plant taxonomy, Botany and Microbiology department, Faculty of Science, Zagazig University, Egypt, [25]. Afterward, the leaves were transferred in sterile bags to the laboratory and gently washed with running water to remove dust. Plant leaves were then prepared following the method described by Petrini [26]. Under aseptic conditions, the leaves were chopped into 3–4 mm × 0.5^–1^ cm pieces with and without midrib. The surface was sterilized with 75% ethanol for one minute. Subsequently, the leaf pieces were soaked in sodium hypochlorite (NaOCl) for 30 s. Later on, the leaf pieces were rinsed with 75% ethanol, followed by three times washing with sterile distilled water. Leaves were finally placed on sterile Petri dishes for fungal isolation.

### Isolation and identification of endophytic fungi

The endophytic fungi were isolated from leaf pieces by placing 5–6 segments in each Petri plate on Potato Dextrose Agar (PDA) processed by streptomycin 100g/mL and penicillin-G 100 units/mL concentrations. The plates were sealed with parafilm and kept in the dark for 4–6 weeks at 28 °C ± 2 °C. After that, fungi growing from plant segments were purified and identified according to morphological methods [27–29]

### Preparation of fungal inocula

The cultures of fungi utilized in this study were inoculated onto Petri dishes using PDA and maintained at 28 ± 2 °C for 7 days to produce spores. The spore suspension was made by cultivating the test fungus on PDA slants for one week at 28 °C till sporulation. The spores were obtained by gently ejecting spores from conidiophores with a sterile inoculation loop after adding 10 mL of sterilized distilled water to the cultures on the agar's surface slants. To eliminate mycelial debris, the spore solution was filtered via four layers of sterile cheesecloth, followed by filtering utilizing Whatman No. 1 filter paper. Adequate dilutions of the stock spore suspension were prepared using sterile 0.1% (v/v) peptone water as a diluent after counting spores by hemocytometer to achieve the target inoculum level of 4 × 10^2^ cells/ mL [30].

### Production of secondary metabolites

Extracellular and intracellular secondary products were produced utilizing Potato Dextrose (PD) liquid media. Following autoclaving, 100 mL of medium was injected with 1 mL of the spore suspension of the examined fungus (4 × 10^2^spores/mL) and kept at 28 °C for 3 weeks under static conditions [21]. In order to isolate secondary metabolites, the new mycelial mat of each fungal strain was pulverized in a mortar using sterile fine sand and chloroform: methanol mixture (2:1, v/v) as a solvent. The resulting mixture was then centrifuged and filtered. Using an air drier, solvent was vaporized, and methanol was introduced to dissolve the metabolites (Gomhuria Co., Egypt) and kept at 5 °C. The broth filtrates were obtained and treated with chloroform: methanol: (2:1, v/v), mobilized properly for 6 h. until complete separation. The mixture was first separated from the filtrates using n-hexane. The base layer was separated. After the extraction of the residues (2 times), the solvent were evaporated completely [31].

### Biological activity of crude secondary metabolites

#### Antioxidant activity

The 1, 1-diphenyl-2-picrylhydrazyl (DPPH) free radicals were used as a reference to measure the endophytic fungal extract’s antioxidant effectiveness [32]. Briefly, DPPH solution (3.8 mL) and 10 μL of every sample (50μg/mL) were combined and maintained for 30 min at 37 °C. At 517 nm, the mixture’s absorbance was measured. The positive reference was ascorbic acid. The investigation was carried out three times. Furthermore, the scavenging effectiveness rate was measured [32].

#### Antimicrobial activity

##### Microorganisms used as indicators

The microorganisms utilized as indicators were sourced from the Regional Center for Mycology and Biotechnology (Al-Azhar University, Cairo, Egypt). The utilized bacterial isolates were as follows: *Staphylococcus aureus* ATCC 25923 (*S.aureus*), *Bacillus subtilis* RCMB 015 NRRL B-543 (*B.subtilis*)*, Enterobacter cloacae* RCMB 001 ATCC 23355 (*E.cloacae*) and *Escherichia coli* ATCC 25922 (*E.coli*). These bacteria were maintained at − 20 °C and were sub-cultured onto Brain Heart Infusion (BHI) broth (Oxoid). Then, they were kept as slope cultures of BHI agar in the refrigerator for one month until used [33, 34]. The utilized fungal strains, such as *Candida albicans* RCMB 005003 ATCC 10231(*C.albicans*) and *Aspergillus fumigatus* (RCMB 002008) (*A.fumigatus*), were maintained as glass beads at – 20 °C. They were then enriched in PDA broth at 30 °C and prepared as slope cultures. The cultures were then stored in the refrigerator for one month until they were used.

##### Primary screening of antimicrobial efficiency of the endophytic fungal crude

Preliminary antimicrobial susceptibility investigations were performed using the agar well diffusion technique [5, 35]. The agar plates were prepared and seeded separately with test microorganisms (PDA for fungi and nutrient agar for bacteria). Under aseptic conditions, plugs with a diameter of 6 mm were placed containing the crude sample (200 μg/mL) and its metabolite (100 µL). In order to facilitate the diffusion of the substances before the growth of microorganisms commenced, the agar plates were kept in the refrigerator for 2 h. As a negative control, a plug filled with solvent was utilized. The antimicrobial activity of the most effective fungal extract was assessed against *B.* s*ubtilis, S. aureus, E. cloacae*, *E. coli*, *C. albicans,* and *A. fumigatus*. The sizes of the inhibition zone diameters (IZD) were calculated [5, 34]. The positive control for bacterial indicators was gentamycin. Through the application of the agar well diffusion technique, the antifungal properties of the fungal sample (200μg/mL) were assessed versus *C. albicans* and *A. fumigatus* and the positive control was ketoconazole. For statistical analysis, each experiment was carried out in triplicate.

##### Thin-layer chromatography (TLC) analysis

To separate the bioactive substances of the crude extracts, TLC was performed using a Silica gel G-60 aluminium sheet (Merck, Germany). Chloroform–methanol (9: 1 v/v) system was used in the development procedures. After reaching the spots at the end line of the TLC, the plates were analyzed using a TLC scanner at Nawah Scientific Research Center, Almokattam, Cairo, Egypt [3].

### Molecular identification of the most active fungal isolate

The most active fungus (A1) was cultured in sterile Petri plates (9 cm diam.) containing 20 mL autoclaved potato sucrose agar (PSA) and then kept for 5 days at 28 °C [37]. The culture was delivered to the Molecular Unit at Assiut University and subsequently placed in the Molecular Culture Collection (AUMC) of the same institution. Prior to being shipped to SolGent Company in Daejeon, South Korea, for sequencing the 18S rRNA gene and conducting polymerase chain reaction (PCR), the DNA was preserved in 1.5 mL autoclaved Eppendorf tubes. Prior to conducting the PCR, the reaction mixture was augmented with ITS1 (forward) and ITS4 (reverse) primers. Primers have these sequences: ITS1 (5′- TCC GTA GGT GAA CCT GCG G -3′) and ITS4 (5′- TCC TCC GCT TAT TGA TAT GC -3′). By the addition of ddNTPs to the reaction mixture, the purified content of PCR was sequenced utilizing the same primers [38]. A comprehensive analysis of the collected sequences was conducted using the Basic Local Alignment Search Tool (BLAST), which is available on the National Centre for Biotechnology Information (NCBI) website. Phylogenetic analysis of the sequences was conducted using MegAlign (DNA Star) software version 5.05.

#### Purification and characterization of the active compounds

The most efficient crude extract was then subjected to instrumental analysis for separation, purification, and identification of the active compounds as follows:

#### GC.MS analysis

The chemical structure of the compounds was analyzed using GC.MS with a Gas chromatography ISQ LT instrument from Thermo-scientific trace 1310 equipped with a single quadrupole mass spectrometer situated at the Regional Centre for Mycology and Biotechnology, Cairo, Egypt. Initially set at 40 °C for 1 min, the GC temperature program was increased by 5 °C per minute until it reached 250 °C for 2 min, and then further increased by 5 °C per minute until reaching 310 °C for 2 min. The separated peaks were detected via the WILEY Database [6].

#### LC-mass analysis

The crude extract with the highest antimicrobial and antioxidant efficiency was injected to electrospray- ionization-mass-spectrometry (LC–MS) positive and negative ion. LC/MS analysis was performed using LC–MS/MS system (Nexera with LCMS-8045, Shimadzu Corporation, Kyoto, Japan)—HPLC (Nexera LC-30AD) equipped with an autosampler (SIL-30AC), temperature-controlled column oven (CTO-20AC) and coupled to triple quadrupole mass spectrometer (Nexera with LCMS-8045, Shimadzu Corporation, Kyoto, Japan). LC–MS was equipped with RP-C18 UPLC column (shimpack 2 mm × 150 mm) possessing 2.7 µm particle size using the following gradient elution (Acetonitrile (ACN), 0. 1% HCOOH in H_2_O) 0–2 min (10% ACN); 2–26 min (10% ACN-80% ACN) and 26–33 (100% ACN) with 0.2 mL/min flow rate. Positive and negative modes were operated during LC–MS/MS with electrospray ionization (ESI). LC–MS/MS data were collected and processed by Lab Solutions software (Shimadzu, Kyoto, Japan) [2].

#### Separation by pure flash chromatography analysis

Flash chromatography was used in order to isolate the desired compound or compounds from crude mixtures. The extracellular metabolites generated from *A. terreus* A1 were purified and separated using a flash chromatography system (PuriFlash 4100 system; Interchim; Montluçon, France). Preparative separations were performed using a flash chromatography system consisting of UV–Vis 190–840 nm, a fraction collector, and a mixing HPLC quaternary pump was used to perform preparative separations. Interchem Software 5.0 was applied to manage and monitor the process. Each band obtained from TLC in the preceding step was prepared as a separate specimen to separate based on their differing affinities for the stationary and mobile phases. Prior to loading the sample dry onto the column containing 12 g of silica column (25 g—flash—NP column 30um), each specimen was dissolved in 50 mL methanol [39]. The systems Hexane–Ethyl acetate (3:1, v/v), solvent (A) and methanol-dichloromethane (1: 9, v/v), solvent (B) were used to run the separation process. After several runs, the final concentrated fraction was obtained. An initial flow rate of 10 mL/min was applied to solvent (A) alone for the first minute, then increased to 60%. Afterwards, the run time was extended to seven minutes while using solvent (B), which was raised to 65%. A hold period of one minute was observed after each 1% increase in the solvent concentration. The eluting fractions can be collected and analyzed further. Thus a desired pure compound can be obtained from the given sample. The purified output resulting from the flash chromatography was applied on TLC, as previously mentioned [8].

#### NMR and FTIR spectral analysis

Nuclear magnetic resonance (NMR) spectral analysis and Fourier-transform infrared (FTIR) spectral analysis were conducted to elucidate the structure of the purified compound [2, 5]. The ^1^H-NMR and C-NMR spectral analyses (Bruker 400 MHz Avance HDIII) was performed at Drug Discovery Center, Research and Development, Faculty of Pharmacy, Ain Shams, Egypt). The sample was dissolved in deuterated methanol. The FTIR analysis was conducted at the Micro-Analytical Centre, Cairo, Egypt, using Bruker Spectrometer FT-IR in the 400–4000 cm^−1^ spectrum with KBr pellets formed into discs under vacuum.

### Biosynthesis of MnNPs and MnNCs

The *A. terreus* cell-free supernatant was used as a biocatalyst for the synthesis of MnNPs from MnO_2_ (Nanotech Company Dream Land, Egypt). According to the procedure reported by [40], 1mM of MnO_2_ was mixed with the *A. terreus* cell-free filtrate. Then, the reaction flask was incubated at 28 ℃ for 3–5 days. Afterwards, MnNPs were detected. Next, the Sono-chemical method was used for the formation of MnNCs [19]. An amount of 1 g of the obtained MnNPs and 1 mL of the purified active compound (HDOCOX) were mixed with 200 mL of deionized water. The mixture was sonicated with amplitude of 85% for 50 min at a frequency of 50 kHz and a cycle length of 0.65, (UP400S, Hielscher, Germany).

### Characterization of both MnNPs and MnNCs

Several analyses were conducted to determine the morphological and physicochemical properties of the biosynthesized MnNPs and MnNCs. Zeta-size calculations were performed to determine the size of MnNPs, and Zeta-potential calculations were performed to determine the stability of MnNPs [21]. Transmission electron microscopy (TEM) and scanning electron microscopy (SEM) were used to determine the morphological features of the MnNPs and MnNCs, following a previous method [21]. Furthermore, the structural characteristics of the synthesized MnNPs and MnNCs were determined using X-ray diffraction (XRD) and energy dispersive X-ray (EDX) techniques, as described in a prior study [7].

### Biological activities of HDOCOX, MnNPs and MnNCs

The biological activities of each HDOCOX, MnNPs and MnNCs were studied as follows:

#### Antioxidant activity

The 1, 1-diphenyl-2-picrylhydrazyl (DPPH) free radicals were used to measure antioxidant effectiveness of the most potent HDOCOX using a positive reference of ascorbic acid as mentioned above [32]

#### Antimicrobial activity

The agar well diffusion technique was performed to determine the antimicrobial efficacy of HDOCOX, MnNPs and MnNCs, and to establish the minimum inhibitory concentration (MIC). Separate stock preparations of HDOCOX, MnNPs and MnNCs (200 μg/mL), were suspended in methanol and kept at a temperature of 5 °C. The antibacterial and antifungal activities were tested as aforementioned against the same strains [5]. To determine the MIC values, HDOCOX, MnNPs, and MnNCs were separately prepared at different dilutions (5, 10, 20, 30, 40, 50, 70, and 100 µg/mL). Each dilution was tested in a separate experiment conducted in triplicates. Upon the completion of one day for bacteria and seven days for fungi, the diameter of the resulting inhibition zones was recorded for further statistical analysis [5, 41].

TEM was conducted to detect the ultrastructure of morphological changes caused by HDOCOX, MnNPs, and MnNCs against *E.coli* and *C. albicans*, which were selected as the most sensitive to the tested compounds. Fresh cultures of *E.coli* and *C. albicans* (10^6^ CFU/ mL) were treated separately with the corresponding MIC and maintained for 4 h. at 37 °C. The control cultures were maintained under identical conditions without any treatment. The cultures were then centrifuged at 4000 rpm for 10 min to isolate the cells of the test strains. Prior to fixation, the cells were thoroughly rinsed with distilled water and then immersed in a solution of 3% glutaraldehyde and potassium permanganate at room temperature for 5 min. The specimens were then left to dry for 30 min. with pure ethanol. Later on, the samples were finally immersed in pure resin, loaded onto TEM copper grids, sliced into thin slices, and stained twice with lead citrate and uranyl acetate to be examined using TEM (JEOL JEM-1010, Tokyo, Japan) [41].

### Cytotoxicity assay

Firstly, cell culture of BNL (Mouse normal liver cells) was obtained from Nawah Scientific Inc., (Mokatam, Cairo, Egypt). Cells were maintained in DMEM media supplemented with 100 mg/mL of streptomycin, 100 units/mL of penicillin and 10% of heat-inactivated fetal bovine serum in humidified, 5% (v/v) CO_2_ atmosphere at 37 °C.

Cell viability was assessed by SRB assay. Aliquots of 100 μL cell suspensions (5 × 10^3^ cells) were in 96-well plates and incubated in complete media for 24 h. Cells were treated with another aliquot of 100 μL media containing drugs at various concentrations. After drug exposure, cells were fixed by replacing media with 150 μL of 10% TCA and incubated at 4 °C for 1 h. The TCA solution was removed, and the cells were washed 5 times with distilled water. Aliquots of 70 μL SRB solution (0.4% w/v) were added and incubated in a dark place at room temperature for 10 min. Plates were washed 3 times with 1% acetic acid and allowed to air-dry overnight. Then, 150 μL of TRIS (10 mM) was added to dissolve protein- bound SRB stain; the absorbance was measured at 540 nm using an Infinite F50 microplate reader (TECAN, Switzerland) [42, 43].

#### Anticancer activity

The purified fungal active compound, MnNPs and MnNCs, was assessed for its anticancer activity, rate of cell viability, and inhibitory action using a colorimetric MTT assay at the VACSERA Tissue Culture Unit according to the procedure described by previous studies [44, 45].For cytotoxicity assay, human hepatocellular carcinoma (HepG-2) cells were treated by trypsin and washed. Then, cells were inoculated in 96-well plates at a final density of 10^4^ cells/100 µL, using RPMI-1640 supplemented with 10% fetal bovine serum (FBS). The plates were incubated with 5% CO_2_ at 37 °C for 24 h. Subsequently, the medium was changed with FBS-free media containing different dilutions of the compounds at twofold serial dilutions (3.90, 7.80, 15.60, 31.25, 62.50, 125, 250, and 500 µg/mL). Following a 48-h incubation period at 37 °C and 5% CO_2_, the wells were rinsed with PBS solvent. The wells were then filled with 50 µL of the MTT reagent (3-(4,5-Dimethylthiazol-2-yl)-2,5-diphenyltetrazolium bromide) 0.5 mg/mL, PBS, and the plates were then left to sit for 4 h. The supernatants were discarded, and the precipitated dark blue formazan crystals were dissolved in 50 µl of dimethyl sulfoxide 99.9% for each well, followed by stirring. Following this, the supernatants were discarded, and the number of live cells was determined by measuring the formazan absorbance at 490 nm using a microplate reader (SunRise, TECAN, Inc., USA). GraphPad Prism software (San Diego, CA, USA) was used to calculate the concentration required to cause detrimental effects on 50% of intact cells [8]. The percentage of viability was calculated as follows:

$${\% Cell viability }=\frac{Mean\,\,O.D\,of\,treated\,\,cells}{Mean\,\,O.D\,of\,untreated\,\,cells}\times 100$$

### Statistical analysis

All experiments were conducted in triplicate. One-way ANOVA was applied to calculate the standard deviations (±SD) [46]. Using the statistical WASP software program 2.0, data were analyzed using the least significant difference (LSD), with a significance level of p < 0.05 for LSD. Specimens’ signs (a.a) indicate a non-significant difference, whereas (a.b) indicate a significant difference [21].

## Results

### Identification of endophytic fungi

A total of 30 endophytic fungi were isolated from the collected medicinal plant leaves (*Moringa oleifera* Lam., *Psidium guava* L.*, Medicago sativa* L.*, Beta vulgarus* L.*,* and *Ocimum basilicum* L.,). As shown in Table (S1), the fungal isolates were initially identified to belong to eight main genera, namely *Aspergillus, Alternaria, Rhizopus, Trichoderma, Penicillium, Fusarium, Cunninghamella,* and *Cladospora*.

### Biological activity of the crude secondary metabolites

#### Antioxidant activity

The antioxidant potential of the crude extracts of the extracellular and intracellular metabolites of the isolated endophytic fungi was detected using the DPPH method. As illustrated in Table (S1) and Figure (S1) and the extracellular secondary metabolites of the fungal isolates had greater antioxidant effects than the intracellular. Of the 30 fungal isolates examined, isolates A1, A2, A4, A7, A8, A13, A20, A27, and A29 exhibited bioactivity in terms of antioxidant activity in both extracellular and intracellular extracts. Furthermore, among the fungal isolates tested, fungal isolate no A1 shows the highest antioxidant activity (% 94.1 ± 0.79). Nevertheless, extracellular outputs (%) 11.8 of fungal isolate A28, was evaluated for weak antioxidant effects.

#### Antimicrobial activity

The analysis revealed that the extracts of most of the isolated endophytic fungus, classified as numbers 1, 2, 3, 4, 5, 7, 8, 10, 12, 14, 15, 17, 18, 20, 21, 22, 24, 27, 29, and 30, exhibited significant antibacterial properties, as shown in Table (S2) and Figure (S2). In comparison to the intracellular extracts, the extracellular extracts of the fungal isolates exhibited a more pronounced antibacterial activity. Conversely, the inhibition zones of intracellular extracts of isolate no A1 against *B. subtilis* and *E. coli* reached 29.4 and 30.3 mm, respectively. On the contrary, every investigated fungal isolate against certain investigated the pathogenic bacteria demonstrated the antibacterial properties of extracellular extracts. Furthermore, the extracellular extracts exhibited inhibition zones of 35.15 mm and 36.13 mm against *E. coli* and *B. subtilis*, respectively. These zones were larger than those detected when gentamicin was used as a control.

The antifungal action of the intracellular extracts of the fungal strains A9, A10, A14, A18, A19, A21, A22, A23, A26, and A28 did not demonstrate any antifungal activity against the tested fungal strains viz. *C. albicans*. Several significant effects were identified from the intracellular extracts of fungal strains, such as A1, A2, A3, A8, A11 and A15, against *C. albicans*. However, some significant effect was identified in the extracellular extracts of fungal isolates codes A1, A2, A3, A4, A8, A10, A11, A15, A17, and A27 against *A. fumigatus* against *C. albicans.* The data presented in Table (S2) and Figure (S3) revealed that A1, the strain of *A. terreus* extract, had a higher level of antifungal effects against *C. albicans* than against *A. fumigatus*. However, the inhibition zone formed by *A. terreus* extract against *C. albicans* and *A. fumigatus* is approximately 40% and 17% larger, respectively, compared to using ketoconazole as a control (Table S2).

#### TLC analysis

The crude extracts of the extracellular products of the isolated endophytic fungi were demonstrated different chemical diversity as given on a TLC silica gel plate (Figure S4).

### Molecular identification of the most active fungal isolate

The isolate *A. terreus* A1 was chosen as the most effective fungus based on the observed antioxidant and antimicrobial properties of the crude extract. Through a comparison of the 18S–28S rRNA sequence, the isolate (A1) was determined to be *Aspergillus terreus*. PCR analysis yielded a DNA band measuring approximately 579 base pairs for *Aspergillus sp*. (Figure S5). The fungus *Aspergillus terreus* was deposited in AUMC under the unique identification number AUMC 15810. The nucleotide sequence was submitted to GenBank with the accession number OR243300. The alignment profile of *Aspergillus sp.* (sample-1) showed unambiguous identification and complete coverage with several strains of the same species, specifically the type material *A. terreus* ATCC1012 with GenBank accession number NR_131276. Thus, the isolate of *Aspergillus terreus* was specifically identified as *Aspergillus terreus*-FM, *Aspergillus terreus* AUMC 15810 (579 letters). It was designated as *A.terreus* with accession no. OR243300, (Figure S6).

### Purification and characterization of active compounds

#### Identified bioactive compounds in GC.MS

As revealed in Table 1 and Figure (S7), *A. terreus* extract demonstrated eleven main compounds with strong antioxidant, antibacterial, and anticancer properties. The bioactive compounds were divided into phenols, flavonoids, alkaloids, alcohol, terpenoids, terpenoids, steroids, and ketone groups. Additionally, the existence of various chemicals, such as aldehydes and oxopentanoic acid, was determined herein using GC.MS. Additionally, the product of *A. terreus* contains heterocyclic and pyrazole compounds, which have significant antioxidant, antibacterial, and anticancer efficiency. The current findings also supported the presence of other bioactive substances, such as ketone and ester, which are known for their antiproliferative properties.

**Table 1:** The compounds of the secondary metabolites of *A. terreus* detected with GC–MS

No	RT	Name & class	Formula & Mol. wt	Parent ion, M	Area	Base peak	Compound structure	Activity
1	5.63	Benzaldehyde (Aldehydes)	C_7_H_6_O (106.0)	106	0.13	105.0 & 7.00		Anesthetic, antibacterial, anticancer, antimutagenic, antipeptic, antiseptic, antispasmodic, antitumor, candidicide, flavor, insecticide, nematicide, pesticide, sedative, termiticide, and tyrosinase inhibitor (Zayed et al. 2014)
2	15.96	3-isopropyl2- methoxy -5- methyl pyrazine (Heterocyclic)	C_9_H_14_N_2_O (166.0)	166	0.46	151		Anti-aging effects (Gueule et al. 2015)
3	17.13	5,6,7,8-tetrahydro- pyrimido[4,5- b]benzo-thiophene -4(3h)-one (Heterocyclic)	C_10_H_10_N_2_OS (206.0)	206	0.43	178		Antimicrobial, anticancer, anti-inflammatory, antioxidant, antitubercular, antidiabetic, anti-convulsant agents. (Keri et al. 2017)
4	19.59	3-methoxy-2- (1-ethyl ethyl)- 5-(2-methyl propyl) pyrazine (Heterocyclic)	C_12_H_20_N_2_O (208.0)	208		166		Antitumor, antifungal, antibacterial activity. (Jaddoa et al. 2016)
5	19.59	Methyl 3-(4- hydroxy-2- nitrophenyl)pr opanoate (Esters)	C_10_H_11_NO_5_ (225.0)	225	5.24	151		Anticancer. (Reta et al. 2012)
6	20.09	3,5-di-tert- butyl-4- methyl- 1h- pyrazole (Heterocyclic)	C_12_H_22_N_2_ (194.0)	194	6.58	179		Antimicrobial, antifungal, antitubercular, antiinflammatory, anti- convulsant, anticancer, antiviral. (Naim et al. 2016)
7	20.33	2-(2-hydroxyhex-1-enyl)-3- methyl- 5,6-dihydropyrazi ne (Heterocyclic)	C_11_H_18_N_2_O (194.0)	194	2.06	137		Fragnance, sedative, carminative, and antiemetic effects. (Peng et al. 2020)
8	21.43	3-(2-hydroxy- 4,4-dimethyl- 6-oxo-1- cyclohexen-1- yl)-4-oxopentanoic acid (acids)	C_13_H_18_O_5_ (254.0)	254	0.72	166		Anticancer. And antimicrobial (NCBI 2022)
9	22.27	3,5-Ditert– butyl-4-ethyl- 1H- pyrazole (Heterocyclic)	C_13_H_24_N_2_ (208.0)	208	72.81	193		Anticancer and antimicrobial (Naim et al. 2016)
10	24.37	5-hydroxy-7- methyl-6- propyl- thiazolo[4,5-b]pyridin-2(3h)-one (heterocyclic	C_10_H_12_N_2_O_2_S (224.0)	224	1.43	195		Anti-inflammatory, Antioxidant. (Chaban et al. 2019)
11	24.68	(3e)-4-(2,3,4-trimethoxy phenyl)-3-buten-2 one (ketone)	C_13_H_16_O_4_ (236.0)	236	9.02	205		Anticancer (NCBI 2022)

#### Identified bioactive compounds in LC–MS/MS

**Fig. 1:**
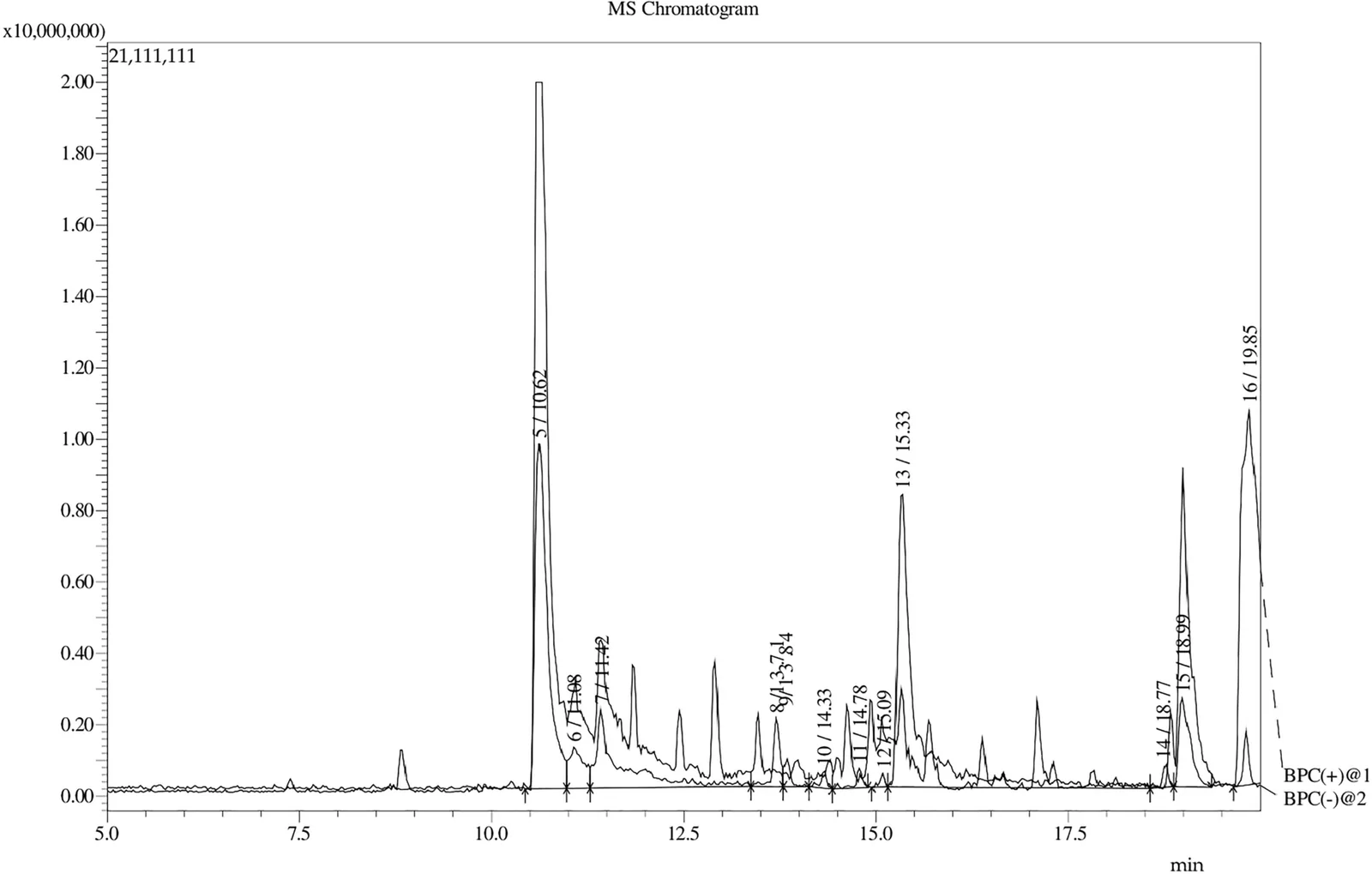
LC–MS of a crude compounds in positive and negative mode

Using the MS/MS spectra and chromatogram of LC–MS/MS, a total of 27 compounds were found in the aqueous extract of *A. terreus* in positive negative mode, (Fig. 1). The positive ions elucidated 11-compounds with molecular masses ranging from 241.20 Da to 502.2 Da. In view of the metabolites composition of the positive ions peaks, isoquinoline alkaloids and indole-alkaloids that predominated the composition of these bioactive compounds. Regarding the negative ion-peaks, 16 compounds of molecular masses in the range 213.20 Da to 775.40 Da were shown (Table 2). In view of the compounds within the negative ions peaks, the majority of compounds were alkaloids, phenolics, terpenoids, steroids, flavonoids, acids and quinones (Table 2). These active compounds were shown to be the main contributors to the antioxidant activity, pharmacology and antimicrobial activities of *A. terreus*. Since several of the other types of chemicals, including fatty acids, aromatic compounds, amino acids, lactones, and heterocyclic ketones, were shown to have pharmacological activities, they also partially contributed to *A. terreus’s* medicinal usefulness.

**Table 2:** Possible metabolites compositions of crude extract of *A.terreus* estimated by MS (ESI–MS) technique

Ions mode/peaks number		Area (%)	Molecular weight	Composition
Positive ion	5,6,7	170,858,074	241.20	(-) TMC 120A C_15_H_15_NO_2_
	8	12,318,112	264.1	Abscisic acid C15H20O4
	9	4,220,218	502.2	Verrucerin A C_27_H_34_ O_9_
	10	2,674,279	296.4	E-8 (3-(oct.2-enoyl)ooxiran-2-yl)octanoic acid
	11	3,482,399	336.15	Cyclopiazonic acid C_20_H_20_N_2_O_3_
	12	1,768,041	353.20	Gliionitrin diacetate C_20_H_16_O_7_
	13	132,892,501	225.15	Sodium3-nitrobenzenesulfonate C_6_H_4_NNaO_5_S
	14	2,976,528	322.15	Asnipyrone A C_21_H_22_O_3_ B hydroxy momilactone
	16	2,034,643,011	336.20	Cyclopiazonic acid C_20_H_20_N_2_O_3_
Negative ion	1	24,936,979	273.18	4-methylprimaquine C_16_H_23_N_3_O
	2	28,816,657,774	239.15	Campyrone C C_12_H_17_NO_4_
	3	27,514,213	399.20	Aspochalasin B C_24_H_33_NO_4_
	4	10,285,101	367.189	Brevianamide C_21_H_25_N_3_O_3_
	5	16,585,062	443.19	Variecolin A C_25_H_30_C1N_3_O_3_
	6	17,287,250	367.15	4-O-methylxanthohumol(1-) C_22_H_23_O_5_
	7	11,849,027	775.40	Aspergilasine C_42_H_47_NO_13_
	8	39,095,800	223.07	Asperopterin A C_8_H_9_N_5_O_3_
	9	11,075,339	213.20	Pc3 C_11_H_16_O_4_ 2,2-Dimethyltrimethylene acrylate
	10	280,401,193	228.25	Resveratrol C_14_H_12_O_3_
	11	7,310,425	236.3	2Hydroxyacoronene C_15_H_24_O_2_
	12	77,725,952	334.3	Palmarumycin CP3 C_20_H_14_O_5_
	13,14	8,711,451	338.1154	Decaspironec C_20_H_18_O_5_
	15	29,007,823	368.25	Variecolin C_25_H_36_O_2_
	16	120,339,025	352.26	MONOLINOLENIN, C_21_H_36_O_4_

#### Flash chromatography

**Fig. 2:**
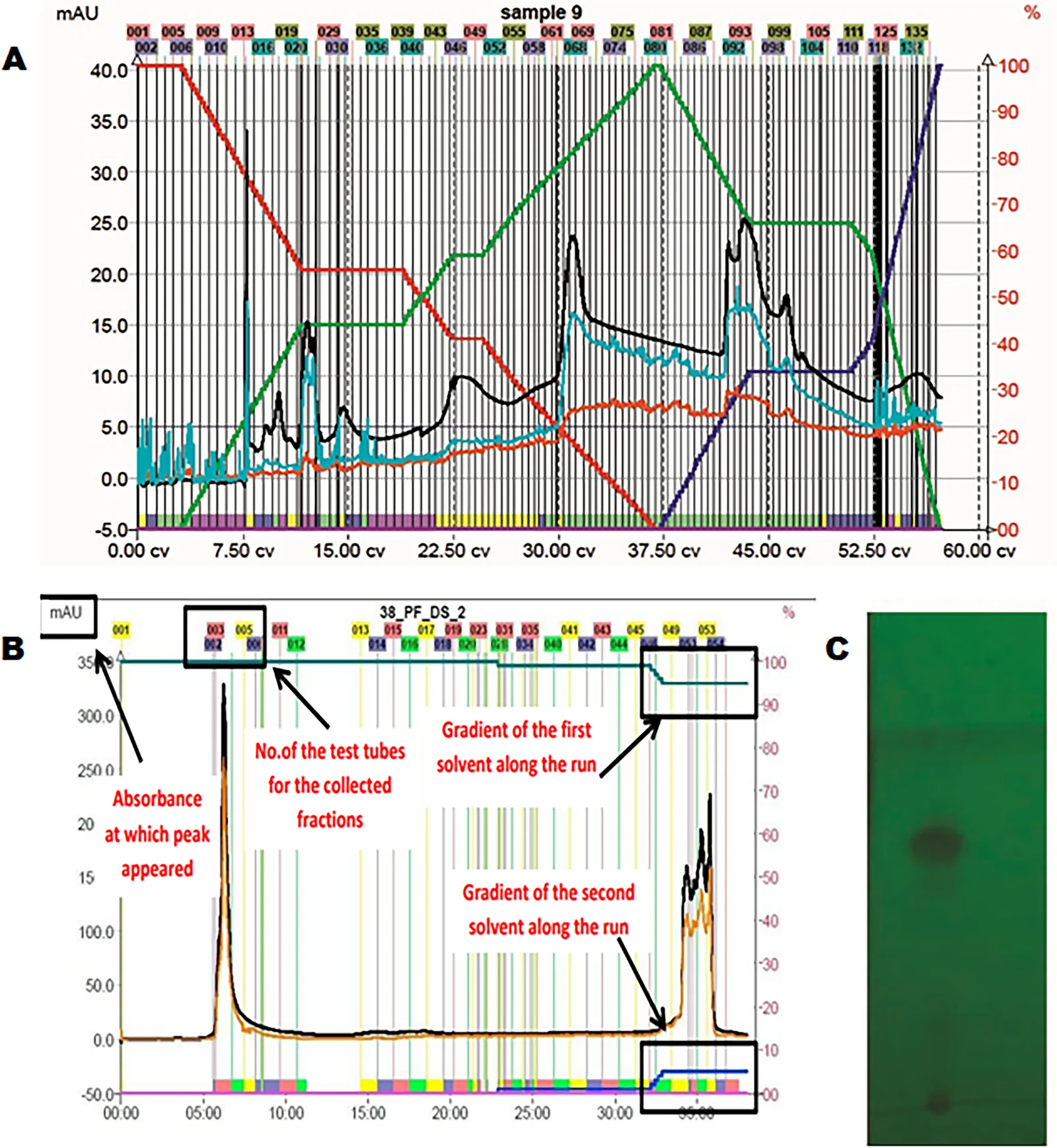
Flash column chromatography analysis: **A** Separation of several compounds including the targeted molecule (large blue peak). of *A.terreus*, **B** Separation chart by running solvent system for target compound of flash chromatography fractions analysis by HPLC shows the desired compound is nearly 100% pure, **C** TLC analysis of purified separated target compound

Flash chromatography was conducted to enhance the separation and purification of the active compounds. Where, it has the power to separate a broad variety of compounds more efficiently than other crude purification techniques. Upon conducting numerous repetitions of several run, it was observed that the separation significantly improved. The final concentrated fraction was obtained, as shown in Table (S3). When the n-hexane extract was analyzed, it emphasized the presence of several products in the secondary metabolites of *A. terreus* A1 (Fig. 2a). The separation was repeated several times, as depicted in Table (S3), using dichloromethane and methanol. Each collected subfraction was repeated and obtained into a signal vial and eluted, as shown in Table (S4). However, there were some distinct peaks for certain subfractions, as demonstrated in Fig. 2b. Consequently, it was assumed that each vial contained an active isolated component. The purification process using flash chromatography yielded a single desired pure organic compound, which appeared as a brownish band on TLC (Fig. 2c), while the other compounds disappeared after repeated purification runs. The desired product was subsequently identified using appropriate instrumental analytical methods, and their potential biological activity was assessed.

#### NMR and FTIR

**Fig. 3:**
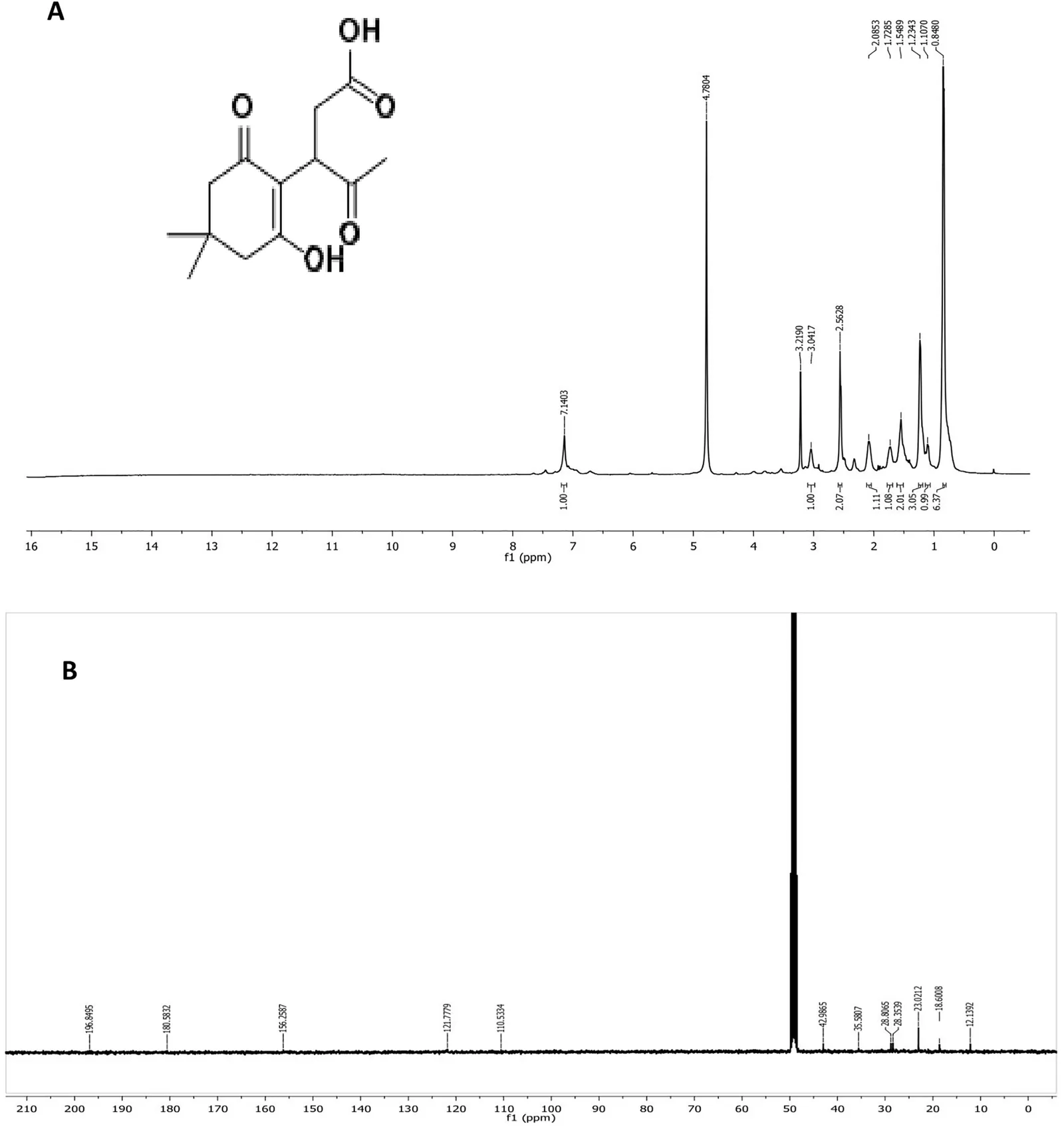
Identification and characterization of the fungal active compound produced from *A. terreus* A1; **A** H-NMR-Spectra, **B** C-NMR spectra of antimicrobial compound produced by *A.terreus*

The chemical structure of the pure extract from *A. terreus* was verified using ^1^H NMR, ^13^C NMR and FTIR analyses. ^1^H NMR (400 MHz, MeOD- d_4_) δ: 7.14 (s, 1H, COOH), 3.04 (bs, 1H, CH), 2.56 (s, 2H, CH_2_), 2.09 (bs, 1H, OH), 1.73 (bs, 1H, CH_2_), 1.55 (s, 2H, CH_2_), 1.23 (s, 3H, CH_3_), 1.11 (bs, 1H, CH_2_), 0.85 (s, 6H, 2CH_3_). This approved the proposal structure, while δ 4.79, and δ 3.21 represent the signal of (deuterated methanol, MeOD- d_4_ (Fig. 3A). Furthermore, ^13^C NMR (125 MHz, MeOD- d_4_) δ spectra have characteristic peaks at δ: 196.8, 180.6, 156.3, 121.8, 110.5, 43.0, 35.6, 28.8, 28.4, 23.0, 18.6, 12.1, while, the signal near δ 50 represents MeOD- d_4_ (Fig. 3B).

**Fig. 4:**
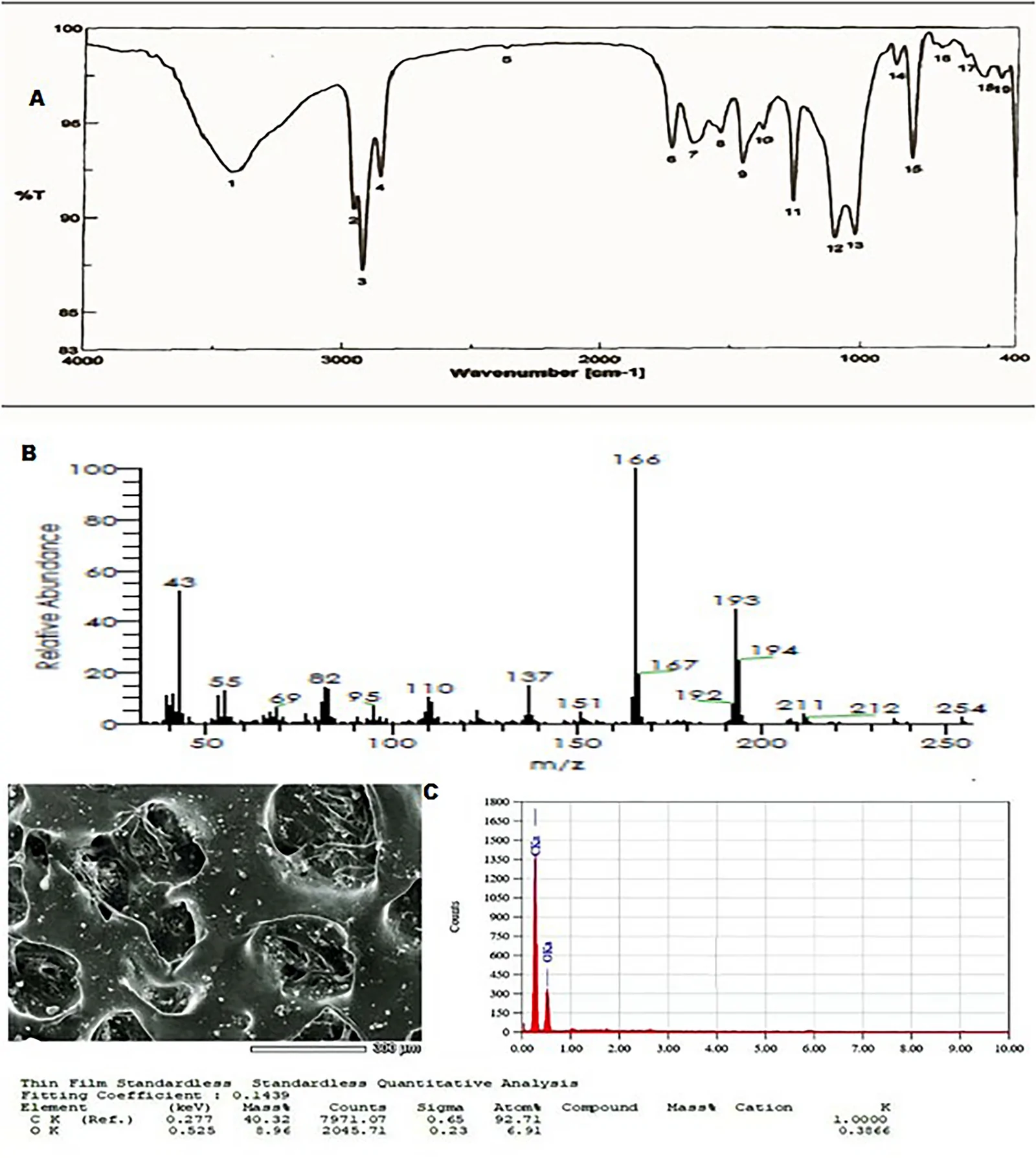
Identification and characterization of the fungal active compound produced from *A. terreus* A1; **A** FTIR spectra, **B** Mass Spectrum of purified 3-(2-Hydroxy-4, 4-dimethyl-6-oxo-1-cyclohexen-1-yl)-4-oxopentanoic acid derivative (HDOCOX), and **C** EDX analysis of purified HDOCOX

The IR spectrum of the extracted compound exhibited distinct peaks that identified the functional groups present in the extract. These peaks included abroad band at 3434 cm^−1^ for OH group of acid, a band at 3428 cm^−1^ for OH of COOH groups, bands at 3428 cm^−1^ for OH of COOH groups, band at 2958, 2924,2855 cm^−1^ for C-H stretching of aliphatic CH_2_ and CH_3_ groups, 1731 cm^−1^ for C=O ester, 1647 cm^−1^ to conjugated carbonyl C=O enolate stretching and 1543 cm^−1^ characterized for C=C as well as band at 1100 cm^−1^ for (–O-) ether linkage.While, the other peaks at 1385–1380 cm^−1^ for C-H bending, bands at 1310–1250 cm^−1^ for C–O stretching of ester, band at 1200 cm^−1^ for O–H bending, bands at 895–885 cm^−1^ for C=C bending and bands at 730–665 cm^−1^ for C=C bending (Fig. 4A).

The total ion chromatogram of mass spectroscopic analysis confirmed the purity of the compound, and a peak of molecular ion at m/z 254 representing its molecular weight and characteristic fragmentation peaks with most stable M/Z ratios at 193, 166, 137 and 43 (Fig. 4B). Thus, GC–MS analysis and identification by NMR, FTIR and mass spectrum confirmed the bioactive compound as (3-(2-Hydroxy-4, 4-dimethyl-6-oxo-1- cyclohexene-1-yl)-4-oxopentanoic acid) with the molecular formula C_13_H_18_O_5_ and has a molecular weight of 254.0 and designated as HDOCOX.

Further, the description of the element on the purified HDOCOX surface was also analyzed using EDX (Fig. 4c). Carbon (40.3%) and oxygen (8.96%) were found in the purified HDOCOX spectrum.

### Characterization of MnNPs and MnNCs

**Fig. 5:**
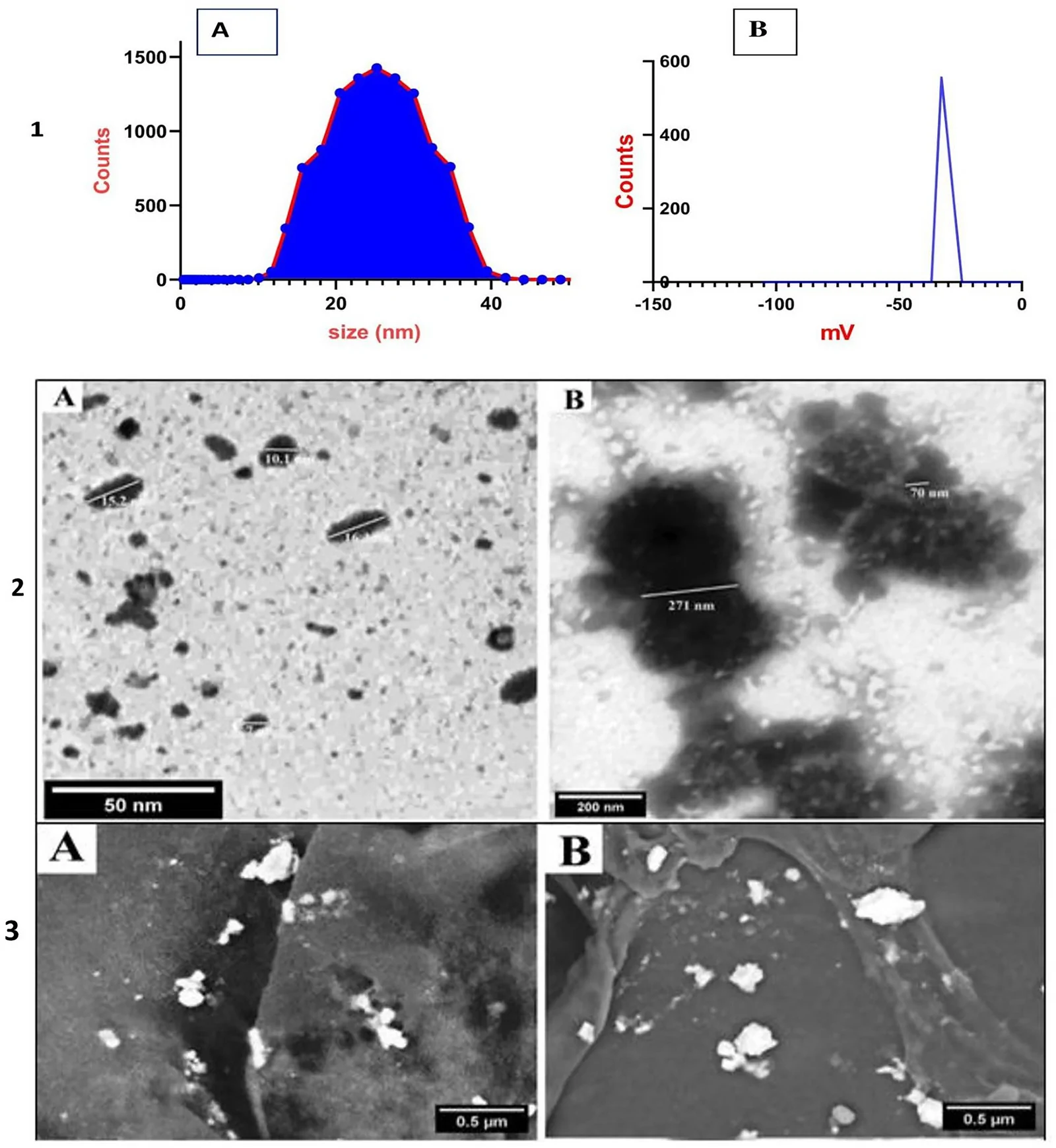
Characterization of MnNPs, and Mn- HDOCOX nanocomposite; **(Panel 1**): (**A**) Zeta size for MnNPs, (**B**): Zeta potential for MnNPs. **(Panel 2**): TEM examination of MnNPs (**A**), and MnNCs (**B**), **(Panel 3):** SEM examination of MnNPs (**A**), and MnNCs (**B**)

MnNPs were biosynthesized by *A. terreus* and have a characteristic peak for diameter at about 25.267 nm (Fig. 5 Panel 1 A). In addition, zeta potential for MnNPs had a characteristic stable peak at -34 mv (Fig. 5 Panel 1 B). The findings revealed that the morphology of the biologically produced MnNPs by *A.terreus* was a spherical rod, and cubic shape for MnNPs by TEM (Fig. 5 Panel 2 A). In addition, the TEM of MnNCs showed sub-rectangular shapes (Fig. 5, Panel 2 B). The SEM of MnNPs and MnNCs showed sub-rectangular shapes (Fig. 5 Panel 3 A & B).

**Fig. 6:**
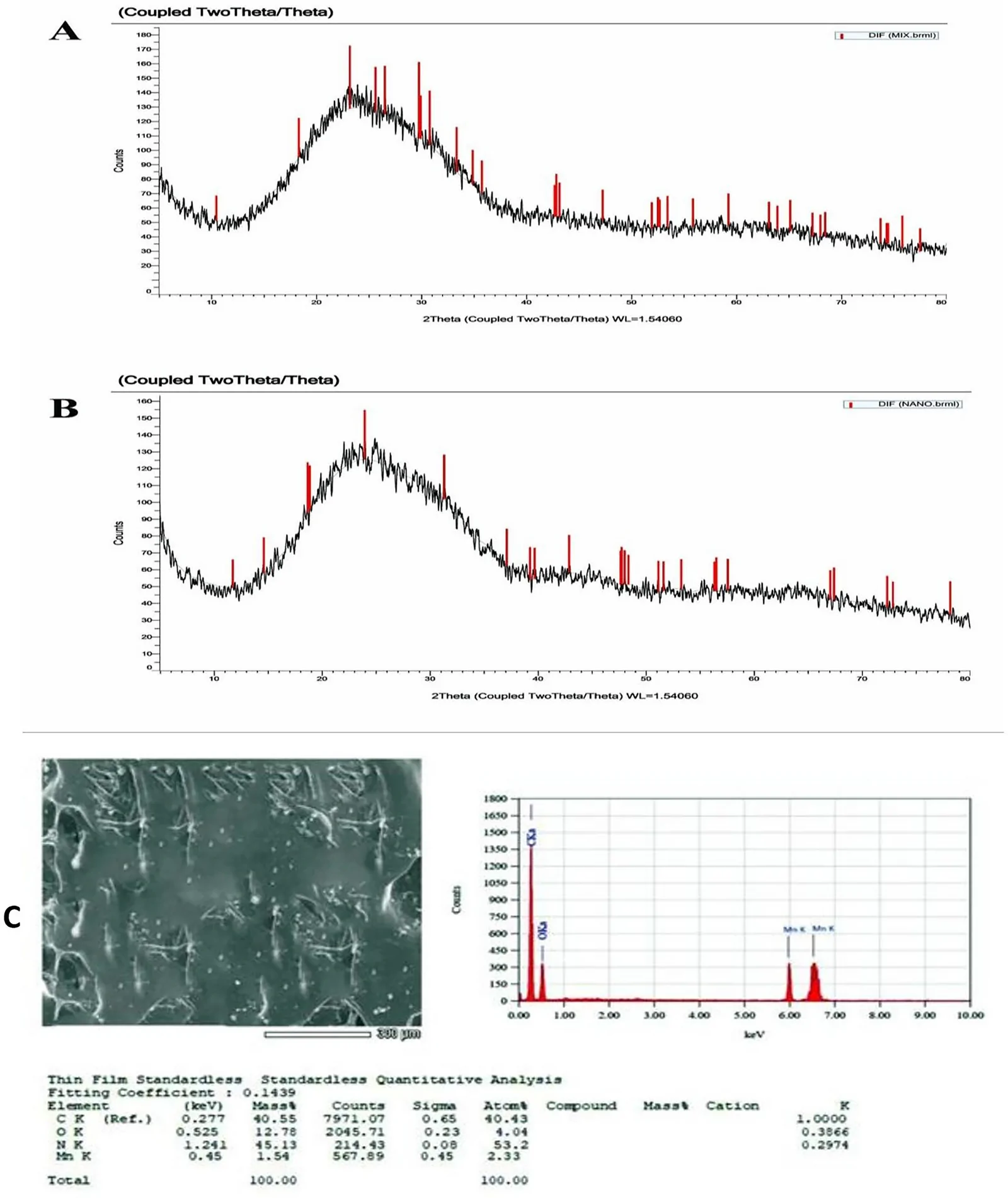
X-Ray (XRD) and EDX analysis. **A** **XRD** for MnNPs, **B** **XRD** for MnNCs. **C** **EDX** analysis of MnNCs

The X-ray diffraction of MnNPs, HDOCOX, and MnNCs are demonstrated in Fig. 6. Characteristic peaks at a 2θ angle of 18.664°, 23.943^0^, 31.306^0^, 37.306°, 42.874° and 47.743°, 48.016°, 67.399° and 78.164° were observed for MnNPs (Fig. 6A). While, MnNCs showed sharp characteristic peaks at 2θ angles 18.321°, 23.182°, 25.618°, 26.511°, 29.760°, 29.917°, 30.777°, 33.340°, 34.884°, 42.841°, 53.442°, 63.896°, 65.119°, and 75.803° indicating cubic lattice of MnNCs (Fig. 6B). The elemental composition on the surface of MnNCs was also analyzed using EDX (Fig. 6C). Whereas carbon (40.55%), Oxygen (12.78%), and Manganese (1.54%) were found in the spectrum of MnNCs.

### Biological activity of HDOCOX, MnNPs and MnNCs

#### Antioxidant activity of HDOCOX

The purified HDOCOX exhibited significant antioxidant activity assessed at 94.25% ± 0.05 with an IC50 value of 5 µg/mL, surpassing both the positive control (ascorbic acid) and the whole extract of the producer strain *A. terreus* A1 (Table S5).

#### Antimicrobial activity

Purified HDOCOX, MnNPs, and MnNCs showed significant antimicrobial effectiveness against all test strains. Among these compounds, the largest diameter of the inhibition zone was observed against *E. cloacae, E. coli,* and *C. Albicans* (Table 3) and (Figure S8). *E. coli* and *C. albicans* were selected for the determination of MICs and detection of the effects of HDOCOX, MnNPs, and MnNCs on the ultrastructure of bacteria and fungi. Comparison of inhibition zones yielded MICs of 20µg/mL for both HDOCOX and MnNPs and 10µg/mL for MnNCs (Table S6).

**Table 3:** Antimicrobial activity of MnNPs, HDOCOX and MnNCs against different pathogenic microbes

Test strains		Diameter of inhibition zone (mm)		
		MnNPs	HDOCOX	MnNCs
Gram + Ve Bacteria	*Staphylococcus aureus*	30.66^b^ ± 0.16	31.96^b^ ± 0.26	38.06^b^ ± 0.40
	*Bacillus subtilis*	29.33^c^ ± 0.37	30.76^c^ ± 0.12	35.43^c^ ± 0.13
Gram − Ve Bacteria	*Escherichia coli*	35.83^a^ ± 0.44	35.30^a^ ± 0.17	42.63^a^ ± 0.26
	*Enterobacter cloacae*	35.56^a^ ± 0.42	36.06^a^ ± 0.06	42.95^a^ ± 0.24
Fungal strains	*Aspergillus fumigatus*	28.96^c^ ± 0.26	20.86^e^ ± 0.82	35.50^c^ ± 0.36
	*Candida albicans*	30.63^b^ ± 0.44	27.50^d^ ± 0.05	36.06^c^ ± 0.29

Means ± SE with different letters in the same columns are significantly different according to Duncan’s multiple range test at p ≤ 0.05

**Fig. 7:**
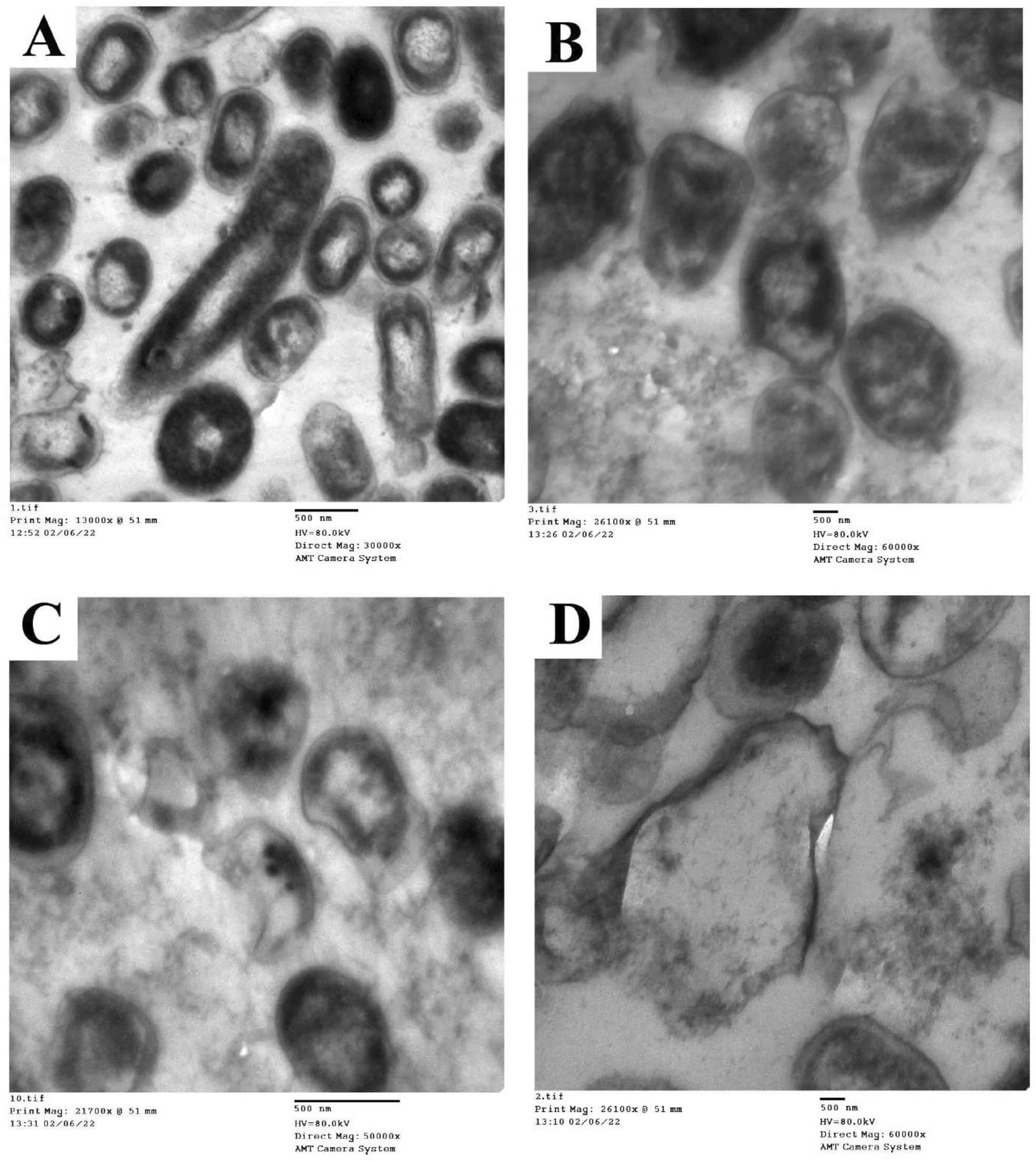
TEM of Antibacterial activity on *E.coli*. **A** *E.coli* control, **B** Effect of HDOCOX on *E.coli*, **C** Effect of MnNPs on *E.coli* **D** Effect of MnNCs with purified substance on *E.coli*

**Fig. 8:**
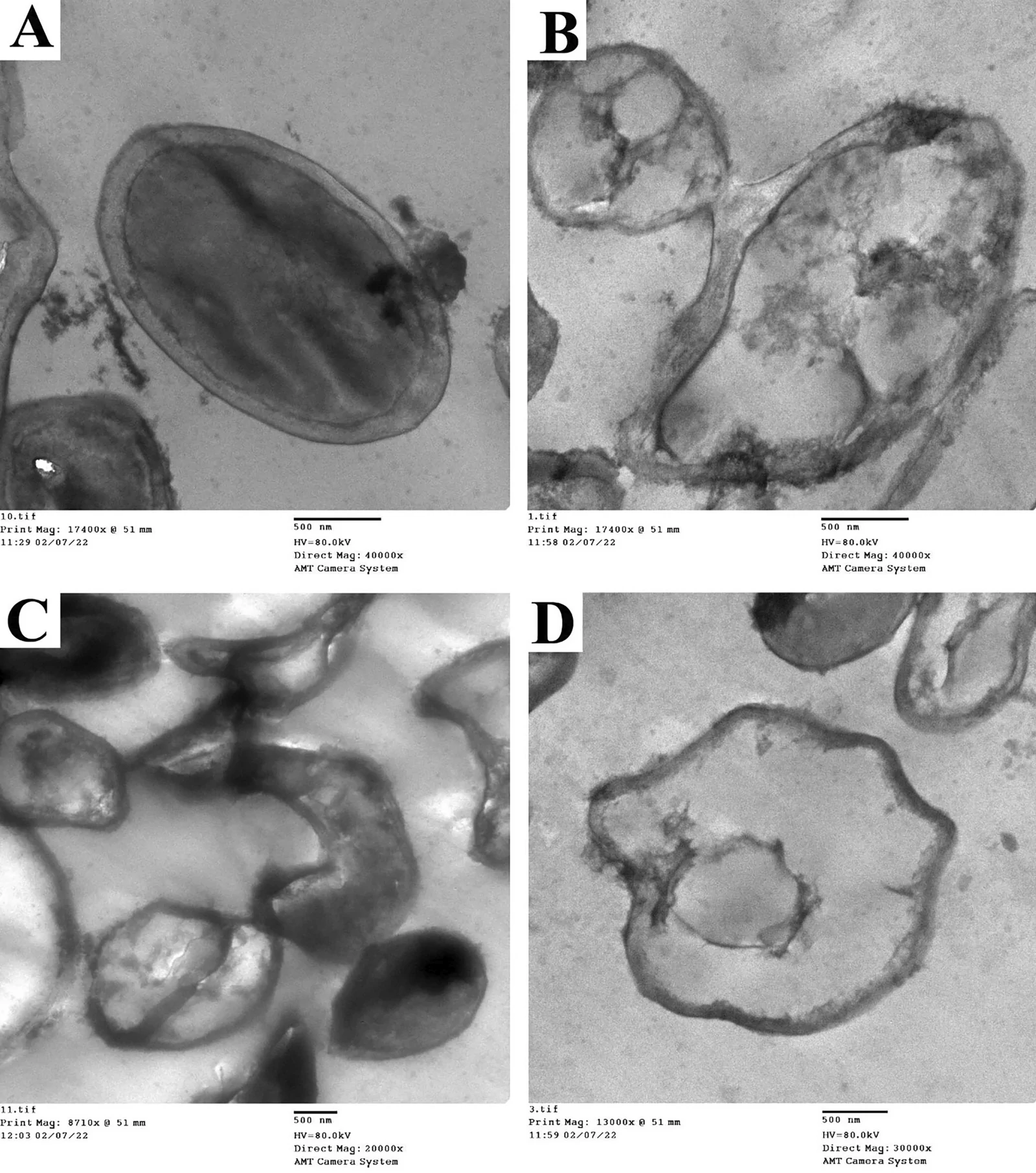
TEM of Antifungal activity on *Candida albicans* **A** *C.albicans* control, **B** Effect of HDOCOX on *C.albicans*, **C** Effect of MnNPs on *C.albicans* **D** Effect of MnNCs with purified substance on *C.albicans*

The TEM micrographs (Figs. 7, 8) showed that the HDOCOX caused a partial disruption of the cell membrane and shrinkage of cytoplasmic material of both *E. coli* and *C. albicans*. In addition, it was observed that the treatment with purified HDOCOX substance, MnNPs, and MnNCs increased or completed cell membrane disruption, which led to the deformation of the treated cells and leakage of intracellular components.

#### Anticancer activity

**Fig. 9:**
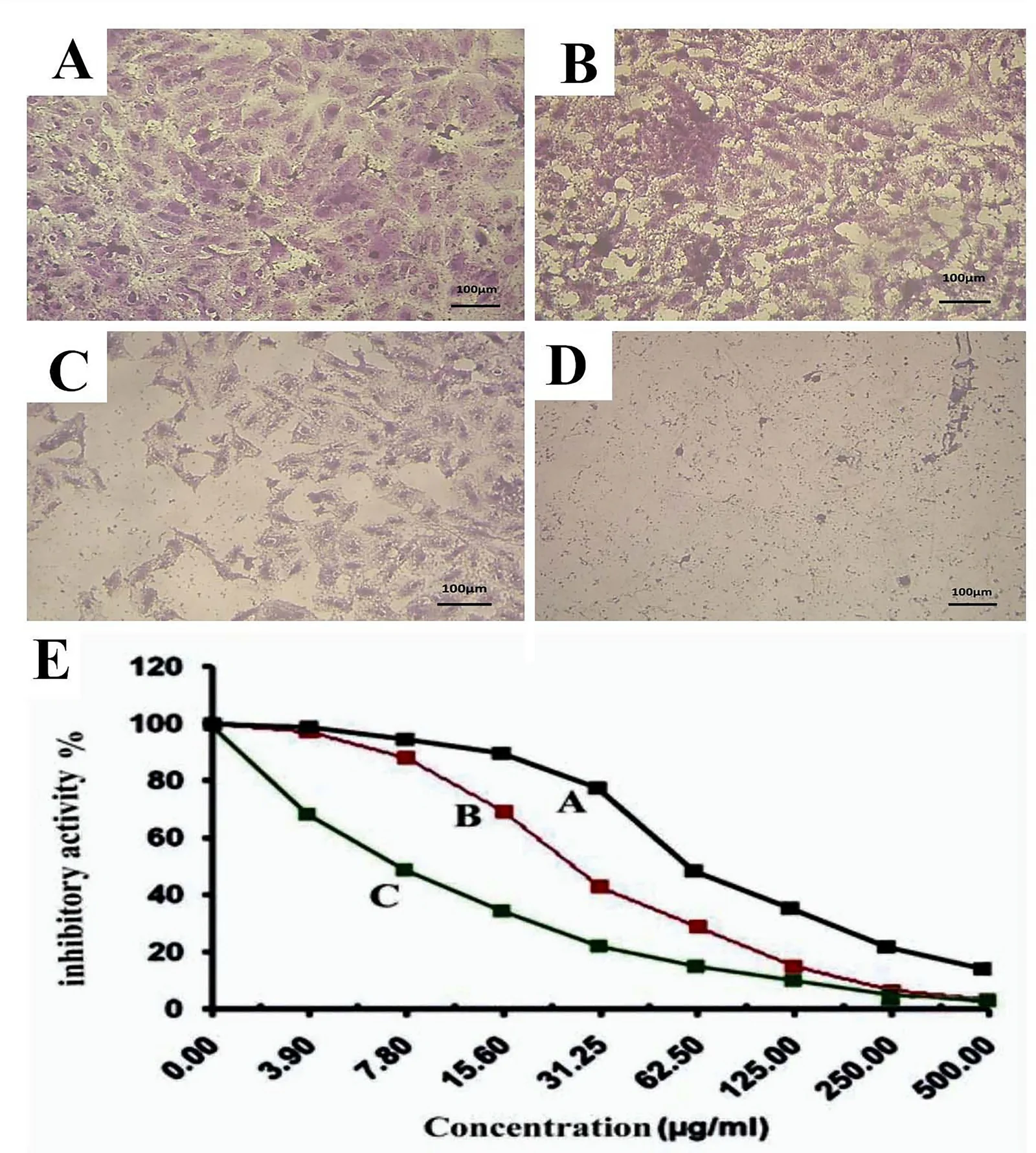
Inhibitory activities on hepatocellular carcinoma cells (HepG cells). **A** HepG cells (control). **B** inhibitory activity of MnNPs on (HepG cells) **C** Inhibitory activity of HDOCOX on HepG cells. **D** Inhibitory activity MnNCs on HepG cells. **E** Determination of cytotoxicity of HepG-2 cell line, A Inhibitory activity Of MnNPS against Hepatocellular carcinoma cells with IC_50_ = 249.19 ± 5.23 µg/mL, **B** Inhibitory activity of HDOCOX from *A.terreus* against Hepatocellular carcinoma cells with IC_50_ = 27 ± 1.1 µg/mL, and **C**: Inhibitory activity of MnNCs against Hepatocellular carcinoma cells with IC_50_ = 7.43 ± 0.1 µg/mL

Firstly, the Preliminary results showed that MnNPs and MnNCs concentrations at 10 and 100μg/mL are non-toxic and the cell viability remain viable and the viability percentage remain constant and safe agent on cell tissue until 100 μg/mL (Figure S9). Later on, The inhibitory effects of HDOCOX, MnNPs, and MnNCs on hepatocellular carcinoma cells were determined and described in Table S7. Moreover, the quantity of cancer cells is progressively decreasing when MnNCs are used, as compared to using standalone purified HDOCOX or MnNPs (Fig. 9A–D). The IC_50_ values for HDOCOX, MnNPs, and MnNCs are 27 ± 1.1, 55.9 ± 1.9, and 7.43 ± 0.1 µg/mL, respectively, as shown in (Fig. 9E).

## Discussion

Given the current AMR rates and increased mortality associated with AMR [5, 6, 47] and various cancer types [48], it is crucial to investigate and develop novel anticancer and antibacterial factors. Therefore, the present study aimed to investigate the endophytic fungi and their secondary metabolites for their antimicrobial and antitumor potential. In addition, the study investigated the biosynthesis of MnNPs and the development of a new nanocomposite for enhanced bioactivity.

Distinct secondary metabolites are attributed to endophytic microorganisms [10]. Several studies demonstrated that the metabolites derived from endophytic fungi can act as immunosuppressants, antibacterial, antifungal agents, and anticancer drugs [24, 49, 50]. Therefore, in this study, we targeted the isolation of endophytic fungi from the leaves of *Moringa oleifera, Psidium guava, Medicago sativa, Beta vulgarus,* and *Ocimum basilicum*. Consistent with several studies [24, 50–52], the intracellular and extracellular secondary metabolites of approximately 70% of the isolated fungi exhibited antimicrobial, antioxidant and cytotoxic activity. These fungal strains included *Aspergillus terreus, Aspergillus oryza, Aspergillus flavus, Penicillium citrinium, Alterutaria alternata, Trichoderma,* and *Rhizopus sp*.

The present study revealed that several fungal secondary metabolites derived from endophytic fungus exhibit antioxidant, antibacterial, and anticancer properties. The secondary metabolite profiles of fungal isolates were characterized. A secondary metabolite is a molecule generated by comparable fungal species, as demonstrated by [51], who illustrated that a secondary metabolite is a chemical substance generated by some fungal species.

Furthermore, the results suggested that antioxidant compounds can eliminate free radicals, which aligns with Ghasemzadeh’s simultaneous study on antioxidant properties [53]. Concerning the DPPH results employed in this investigation, it was found that *A. terreus* had a significant antioxidant capacity of 94.1% ± 0.79 with IC50 at 5µg as opposed to the IC50 values (5 µg) of ascorbic acid that was applied as a reference antioxidant 94.25a % ± 0.05 [54]. Fungi may include a variety of substances with antioxidant properties [55].

The endophytic fungi presented exhibited potent antimicrobial activity towards *E. cloacae, S. aureus, B. subtilis, E.coli, A. fumigatus,* and *C.albicans.* Previous studies using various endophytic isolates have shown that endophytic fungi can produce antibacterial metabolic compounds [47, 56]. The bioactivity results of this work revealed the presence of several bioactive chemicals in the metabolites of the fungal isolates tested. The substantial metabolite richness and diversity of these endophytic fungi in this study unequivocally demonstrate their potential applications.

The *A. terreus* isolate was the strongest isolate and had the highest levels of antioxidant and antibacterial capacity among the indicator microorganisms examined. Consequently, the antimicrobial strain *A. terreus* was subjected to identification tests regarding morphological and molecular features by sequencing the 18S rRNA gene [23].

Since the extracellular metabolites of *A. terreus* A1 exhibited antioxidant and antimicrobial activity more than the intracellular metabolites, they were subjected to several analyses to identify the active compounds. The GC.MS analysis of *A. terreus* A1 secondary metabolites revealed the presence of several compounds, including phenol, alcohol, terpenoid, aldehyde, ketone acid, and others that are known for their antibacterial, antioxidant, and anticancer properties. These findings agree with other prior studies [57, 58]. Additionally, In addition, this study has identified certain chemicals, such as oxopentanoic acid and aldehydes detected using Gc.mass analysis, which can act as both anticancer and antibacterial agents [59]. Ketones and esters, which are typically more hydrophobic and positively charged, can interact electrostatically with the components of bacterial cells. This results in the production of completely de-energized dead cells and a loss of cell viability [6, 60]. *A. terreus* extract contains heterocyclic and pyrazole substances, which have significant antibacterial, antioxidant, and anticancer efficiency [61–63]. By interacting with the cells’ electrophiles or nucleophiles, heterocyclic molecules discovered here by GC–MS analysis suppress pathogenic bacterial cells. This results in the suppression of DNA synthesis, which kills the cells [6, 64]. Moreover, the current findings are consistent with prior reports that other bioactive chemicals such as pyridine, ester, pyridine, and ketone were detected. These compounds are known for their antiproliferative properties [59, 64–67]. Furthermore, The LC.MS analysis of *A. terreus* extract revealed the presence of several compounds at positive ions, including isoquinone- alkaloids and indol-alkaloids that are known for their antimicrobial, antioxidant, and anticancer properties [68]. In addition, this study has identified certain chemicals compounds at negative ions, such as phenols, quinones, fatty acids and terpenoids that are known for their antimicrobial, antioxidant, and anticancer. These findings agree with other prior studies [69–72].

Flash chromatography techniques can provide large amounts of ultra-pure compounds with low time consumption [39]. In this study, a successful separation was obtained by using Pure flash C30 [39, 73]. A comparative analysis of the flash chromatography method with other analytical techniques, such as HPLC and column chromatography, demonstrated the ability to collect higher amounts of purified materials with a shorter duration and solvent [39, 74, 75]. In the present study, TLC was performed, and several bands referring to the variable compounds were revealed, followed by flash chromatography for each of the resulting TLC bands to undergo multiple runs to get high-purity compounds. Since the potential of purification solvents varies depending on the chemical content of the active components [76], the bioactive compounds from the *A. terreus* extract were purified using a two-step technique, which included solvent extraction and flash chromatography. Both non-polar and polar solvents were employed to form hydrogen bonds with the target material during the extraction process. The extraction takes place due to the presence of characteristic variations that allow the chemical potential to form hydrogen bonds with various solvents [5]. This is feasible since the separated molecules have both non-polar and polar parts. In accordance with the procedures and the obtained results of [8, 77], sharp fractions containing the bioactive compounds were refined with non-polar (n-hexane) and polar (ethyl acetate) solvents, as well as dichloromethane.

Due to the importance of the chemical structure of the bioactive compounds, several analysis were employed. Based on the data obtained from FTIR, NMR, and EDX analyses, the active product was described as a keto-acid derivative named 3-(2-Hydroxy-4, 4-dimethyl-6-oxo-1-cyclohexen-1-yl)-4-oxopentanoic acid. It was designated as HDOCOX with a chemical formula of C_13_H_18_O_5_. The antimicrobial, anticancer, and antioxidant activity of other related 4-oxopentanoic acid derivatives [78–80] can be attributed to the presence of ketone and ester groups, which are typically more hydrophobic and positively charged. The presence of such groups within the compound gives it the capacity to interact with the components of bacterial cells electrostatically, which results in loss of cell viability [6, 60].

The therapeutic efficiency of nanoparticles and the potential for their biosynthesis have been extensively documented in the literature [24, 81, 82]. Several studies emphasized that metal-oxide nanoparticles have authentic antibacterial activity due to their small diameter, which can permeate the microbial cell membrane, causing toxicity and cell damage [23]. Therefore, following the identification of a pure bioactive compound in the secondary metabolites of *A. terreus* A1, we focused on utilizing the cell-free extract of the same fungus in biosynthesizing MnNPs. The biosynthesized MnNPs in the present study exhibited a good size at about 25.267 nm using DLS with high stability at -34mv using Zeta potential similar to that reported in the previous study [83] that confirmed the MnNPs were irregularly variable shapes with size ranging from 36.6 to 112.0nm. Also, TEM micrographs revealed the formation of MnNPs of variable shapes with the absence of agglomeration, and the MnNPs were uniformly dispersed throughout the solution. This could be attributed to the varied compounds present in the fungal product, which could serve as capping and agglomeration-preventing agents [23]. In addition, the previous study demonstrated that the synthesized MnNPs are 17–35 nm and spherical in shape using a characterization methods with DLS and TEM analysis [84]. Moreover, the biosynthesized MnNPs herein were mixed with the fungal bioactive acid (HDOCOX) to create a novel nanocomposite that is anticipated to exhibit enhanced bioactivity. The XRD analysis demonstrated the presence of a distinctive cubic lattice and peaks of MnNPs in the novel nanocomposite as reported in the previous study [85]. In addition the previous study by Dhoble and Kulkarni [86] exhibited that the biosynthesized MnNPs is with crystalline in nature and this is agreement with our results. The present work investigated the nanocomposite of purified *A.terreus* extract with MnNPs, focusing on the EDX surface area with Oxygen (12.78%), and Manganese (1.54%). These findings are consistent with the results reported in a previous study [7]. On the other hand, the previous study exhibited that the elemental compositions of MnNPs were 67.41% Manganese and 32.59% Oxygen [86].

The current study aimed to evaluate the antimicrobial and anticancer efficacy of purified *A. terreus* extract, MnNPs, and hybrid nanocomposites against pathogenic bacterial and fungal species. Prior research has substantiated the notion that metal oxide nanoparticles emphasize their essential role as antibacterial agents [23]. In the current study, MnNCs antimicrobial activities were found to be significantly higher than those of solo MnNPs or HDOCOX against *E. coli* and *C.albicans*. This finding agrees with Sahu [87], who demonstrated that when two substances are mixed together, a positive interaction results, and their combined inhibitory impact is larger than the sum of their separate impacts. Also, a prior investigation discovered that the combination of silver nanoparticles with voriconazole or fluconazole was successful in curing drug-resistant *C. albicans*. In addition, previous literature demonstrated the MnNPs’ antibacterial efficacy against *E. coli, K. pneumoniae*, and *P. aeruginosa,* either by themselves or in conjunction with various antibiotics at 12, 14, and 18 mm. The huge surface area and nanosize of MnNPs contribute to their synergistic impact with antibiotics. This allows the antibiotic materials to be more easily integrated and supplied into the cells, as well as disseminated into transfer channels and cell walls, facilitating the release of metabolites [88, 89]. Previous studies also indicated that silver load with MnO_2_ disturbed the cell of *E.coli* [90]. The present work demonstrates that MnNPs exhibit a high degree of simplicity in their binding and ability to seamlessly penetrate the structures of microbes, including blastospores and cell walls. Furthermore, their efficacy in promoting yeast morphogenesis is consistent with the findings of a prior study [81].

Furthermore, previous studies have shown that the inhibitory efficacy of fungal extract can be attributed to the action of purified chemicals and organic acids [91]. The isolated fungal compound positively amino acid moieties showed pore creation and electrostatic characters within cell walls, which resulted in cellular electrolyte loss and ultimately caused lysis of cells [43]. They cross cell membranes, interact with specific molecules, or prevent the creation of necessary substances like glycan and chitin or cell wall formation. Lipopolysaccharides and lipoteichoic acid, which are negatively charged components of microbial cell membranes, are disrupted and depolarized as a result of their cationic amino acid residues and ability to form dimers. Additionally, organic acids such as oxopentanoic acid have been demonstrated to have antibacterial properties by reducing pH levels developing an acidic media that inhibits the growth of pathogenic *C.albicans* and *E. coli* [92]. Therefore, the nanocomposite showed increased antimicrobial properties.

Consistent with earlier research findings [17, 48, 93], the current study emphasized that fungal products can successfully serve as a sequential source for the isolation of new antiproliferative cancer drugs. The MTT assay showed that the fungal HDOCOX possessed in vitro antitumor activity towards the HepG2 cell line. Furthermore, similar to the antimicrobial testing, this assay showed that the cytotoxicity of HDOCOX greatly increased when the HepG2 were treated with MnNCs. Therefore, our findings indicated the synergism between MnNPs and the fungal metabolite HDOCOX in terms of both antimicrobial and anticancer activity, which can be utilized in the development of a novel generation of antimicrobial and antitumor medications.

## Conclusion

Three anticancer, antimicrobial, and antioxidant compounds derived and synthesized from endophytic fungus are presented in this study. These three novel agents consist of the recently developed 4-oxopentanoic acid derivative (HDOCOX) derived from the secondary metabolites of the endophytic fungus *A. terreus*, the MnNPs biosynthesized from the cell-free extract of the same fungus, and a novel nanocomposite of MnNCs. The three agents demonstrated substantial antimicrobial efficacy in comparison to the positive controls. In addition, they efficiently inhibited the proliferation of hepatic cellular carcinoma in vitro, and both HDOCOX and MnNCs demonstrated remarkable antioxidant activity. Therefore, this study focuses on the abundance of bioactive chemicals generated by endophytic fungus that can effectively address the health threat of AMR and the drawbacks of the common anticancer. Additionally, this study demonstrated the potential use of nanoparticles in combination with fungal metabolites to improve their bioactivity.

## Supplementary Information

**Additional file 1: Table S1** Endophytic fungi isolates, their preliminary identification, CF, and the antioxidant potential of their intracellular and extracellular extracts (50 µg/mL). **Table S2** Antimicrobial activity of fungal extracts of endophytic fungi on different pathogenic microorganisms. **Table S3** Collection Table of final concentrated fraction obtained by flash chromatography. **Table S4:** Elution steps of sub-fractions. **Table S5:** DPPH scavenging activity of HDOCOX with IC_50_**. Table S6:** MICs of Mn-Nps, HDOCOX, and Mn-NPs-HDOCOX nanocomposite against *E.coli* and *C. albicans***. Table S7** Detected cytotoxicity at different concentrations of MnNps, HDOCOX and MnNPs-HDOCOX nanocomposite against HepG-2 cell line. **Figure S1** Endophytic fungi isolates, their antioxidant potential of their intracellular and extracellular extracts (50 µg/mL). **Figure S2** Antibacterial effect of most active extracellular extracts of fungal isolates codes A1, A2, A3, A4, A5, A7, A8, A10, A12, A14, A15, A17 and A18 on *E. coli*, *E. cloacae, B. subtilis* and *staph. aureus.* **Figure S3** Antifungal effect of most active extracellular extracts of fungal isolates codes A1, A2, A3, A4, A8, A10, A11, A15, A17, A18 and A27 on *A. fumigatus and C. albicans***. Figure S4** TLC chromatogram of 30 fungal secondary metabolites extracts. **Figure S5** ITS sequences nucleotides region of rDNA of the fungal sample isolated in the present study (*Aspergillus terreus* AUMC15810). **Figure S6** Phylogenetic tree based on ITS sequences of rDNA of the fungal sample isolated in the present study (*Aspergillus terreus* AUMC15810 with accession no.OR243300, arrowed) aligned with closely related strains accessed from the GenBank. This strain showed 100% identity and 100% coverage with several strains of the same species including the type material *A. terreus* ATCC1012 with GenBank accession no NR_131276. *Penicillium chrysogenum* represents an outgroup strain. A = Aspergillus, P = Penicillium. **Figure S7** GC–MS chromatogram of secondary metabolites of *A.terreus*. **Figure S8** Antimicrobial effect of (1): MnNCs, 2): MnNPs, 3): HDOCOX on *E. coli* and *C. albicans.* **Figure S9** Cytotoxicity assay of MnNPS and MnNCs on normal cell line (Mouse normal liver cells) as Preliminary test for determination the cell viability.

## Data Availability

No datasets were generated or analysed during the current study.

## Abbreviations

*Aspergillus terreus*: *A. terreus*
DPPH: 1, 1-Diphenyl-2-picrylhydrazyl
CD: Czapek–Dox
FTIR: Fourier transform infrared
EDX: Energy dispersive X-ray
SEM: Scanning Electron Microscope, electrospray- ionization-mass-spectrometry (LC–MS)
TEM: Transmission Electron Microscope
