# Correction: Antimicrobial and antiproliferative activity of biosynthesized manganese nanocomposite with natural derivative originated by endophytic *Aspergillus* *terreus*

**DOI:** 10.1186/s12934-026-03117-4

**Published:** 2026-10-09

**Authors:** Nashwa El-Gazzar, Reem Farouk, Nervana S. Diab, Gamal Rabie, Basel Sitohy

**Affiliations:** 1 Botany and Microbiology Department, Faculty of Science, Zagazig University, Zagazig, 44519 Egypt; 2 Biochemistry Department, Faculty of Science, Mansoura University, Mansoura, 35511 Egypt; 3 Department of Clinical Microbiology, Infection and Immunology, Umeå University, 90185 Umeå, Sweden; 4 Department of Diagnostics and Intervention, Oncology, Umeå University, 90185 Umeå, Sweden

**Correction: Microbial Cell Factories (2025) 24:37**

**https://doi.org/10.1186/s12934-025-02651-x**

In article [1], we identified an issue requiring editorial correction

1. **Terminology Correction for HDOCOX**: During final drafting, the descriptor “amide derivative” was mistakenly included in the Title, Abstract, and one FTIR section. The correct identity of the compound is a **4-oxopentanoic acid derivative (HDOCOX)** with formula C_13_H_18_O_5_. This is consistently described in the Discussion, Conclusion sections and analytical Characterization that based on the data obtained from FTIR and NMR. The active product was described as a keto-acid derivative, where its biological activity is attributed to ketone and carboxyl/ester groups. The FTIR band at 1647 cm^–1^ should be assigned to conjugated carbonyl C=O enolate stretching of the dimedone core, not an amide carbonyl.

To harmonize terminology and ensure clarity, we respectfully request the following editorial updates:

- **Article Title**,** Abstract**: Replace “amide derivative” with “natural derivative” throughout the text.
- **FTIR result section**: Correct the functional group assignment at 1647 cm⁻¹ to conjugated carbonyl C=O enolate stretching rather than amide carbonyl.
