# Supplementary material for: Antimicrobial and antiproliferative activity of biosynthesized manganese nanocomposite with amide derivative originated by endophytic Aspergillus terreus
Source: Microb Cell Fact. 2025 Feb 4;24:37. doi: 10.1186/s12934-025-02651-x (PMC11796263; doi:10.1186/s12934-025-02651-x)
Supplement: Supplementary file 1 — Additional file 1: Table S1 Endophytic fungi isolates, their preliminary identification, CF, and the antioxidant potential of their intracellular and extracellular extracts (50 µg/mL). Table S2 Antimicrobial activity of fungal extracts of endophytic fungi on different pathogenic microorganisms. Table S3 Collection Table of final concentrated fraction obtained by flash chromatography. Table S4: Elution steps of sub-fractions. Table S5: DPPH scavenging activity of HDOCOX with IC50. Table S6: MICs of Mn-Nps, HDOCOX, and Mn-NPs-HDOCOX nanocomposite against E.coli and C. albicans. Table S7 Detected cytotoxicity at different concentrations of MnNps, HDOCOX and MnNPs-HDOCOX nanocomposite against HepG-2 cell line. Figure S1 Endophytic fungi isolates, their antioxidant potential of their intracellular and extracellular extracts (50 µg/mL). Figure S2 Antibacterial effect of most active extracellular extracts of fungal isolates codes A1, A2, A3, A4, A5, A7, A8, A10, A12, A14, A15, A17 and A18 on E. coli, E. cloacae, B. subtilis and staph. aureus. Figure S3 Antifungal effect of most active extracellular extracts of fungal isolates codes A1, A2, A3, A4, A8, A10, A11, A15, A17, A18 and A27 on A. fumigatus and C. albicans. Figure S4 TLC chromatogram of 30 fungal secondary metabolites extracts. Figure S5 ITS sequences nucleotides region of rDNA of the fungal sample isolated in the present study (Aspergillus terreus AUMC15810). Figure S6 Phylogenetic tree based on ITS sequences of rDNA of the fungal sample isolated in the present study (Aspergillus terreus AUMC15810 with accession no.OR243300, arrowed) aligned with closely related strains accessed from the GenBank. This strain showed 100% identity and 100% coverage with several strains of the same species including the type material A. terreus ATCC1012 with GenBank accession no NR_131276. Penicillium chrysogenum represents an outgroup strain. A = Aspergillus, P = Penicillium. Figure S7 GC–MS chromatogram of secondary metabolites of [file 12934_2025_2651_MOESM1_ESM.docx]

**Antimicrobial and Antiproliferative activity of biosynthesized manganese nanocomposite with amide derivative originated by endophytic *Aspergillus terreus***

**Nashwa El-Gazzar^1,^ Reem Farouk^1^, Nervana S. Diab^2^, Gamal Rabie^1^, Basel Sitohy^3,4*^**

^1*^ Department of Botany and Microbiology, Faculty of Science, Zagazig University, Zagazig 44519, Egypt.

^2^ Department of Biochemistry, Children Hospital, Faculty of Medicine, Mansoura University, Mansoura, 35511, Egypt.

^3^ Department of Clinical Microbiology, Infection and Immunology, Umeå University, 90185 Umeå, Sweden

^4^ Department of Diagnostics and Intervention, Oncology, Umeå University, SE‑90185 Umeå, Sweden, Umeå University, Umeå 90185, Sweden

**^3,4^** [**basel.sitohy@umu.se**](mailto:basel.sitohy@umu.se)**, ^1^, ns_elgsazzar@zu.edu.eg**

**Table S1** Endophytic fungi isolates, their preliminary identification, CF, and the antioxidant potential of their intracellular and extracellular extracts (50 µg/mL).

| **Source** | **Isolate code** | **Preliminary identification** | **CF (%)** | **DPPH scavenging (%)** | |
| --- | --- | --- | --- | --- | --- |
|  |  |  |  | **Extracellular extract** | **Entracellular extract** |
| *Moringa oleifera* | A1 | *Aspergillus terreus* | 42.9 85 | 94.1±0.79 | 77.8±0.21 |
|  | A2 | *Aspergillus* sp. | 57.1 | 46.3±1.35 | 18±0.23 |
|  | A3 | *Aspergillus* sp*.* | 14.3 | 22.9±0.18 | - |
|  | A4 | *Rhizopus* sp*.* | 85.7 | 74.2±0.99 | 29.6±0.44 |
|  | A5 | *Alternaria* sp. | 14.3 | - | - |
| *Psidium guava* | A6 | *Alternaria* sp. | 14.3 | - | - |
|  | A7 | *Trichoderma* sp | 28.6 | 73.5±1.61 | 34.6±0.73 |
|  | A8 | *Alternaria* sp | 14.3 | 34.1±0.6 | 62.7±0.45 |
|  | A9 | *Cladosporium* sp. | 57.1 | - | 84.3±0.95 |
|  | A10 | *Alternaria* sp. | 14.3 | - | - |
|  | A11 | *Aspergillus* sp*.* | 28.6 | - | - |
|  | A12 | *Penicillium* sp*.* | 28.6 | - | - |
| *Medicago sativa* | A13 | *Aspergillus* sp. | 14.3 | 81.8±0.41 | 16±1.32 |
|  | A14 | *Cunninghamella* sp. | 28.6 | - | - |
|  | A15 | *Alternaria.* sp. | 14.3 | 63.5±0.94 | - |
|  | A16 | *Aspergillus* sp. | 57.1 |  | 15.7±1.56 |
|  | A17 | *Alternaria* sp. | 42.9 | - | - |
|  | A18 | *Aspergillus* sp. | 28.6 | 64.8±0.86 | - |
| *Beta vulgarus* | A19 | *Alternaria* sp. | 28.6 | 46.3±0.58 | - |
|  | A20 | *Alternaria* sp. | 57.1 | 67.4±0.59 | 77.4±0.8 |
|  | A21 | *Alternaria* sp. | 42.9 | 60.1±0.95 | - |
|  | A22 | *Penicillium* sp. | 71.4 | 20.3±0.61 | 64.6±0.26 |
|  | A23 | *Fusarium* sp. | 14.3 | - | - |
|  | A24 | *Aspergillus* sp. | 14.3 | - | - |
|  | A25 | *Fusarium* sp. | 100 |  |  |
| *Ocimum basilicum* | A26 | *Fusarium* sp. | 14.3 | 58.2±0.65 | - |
|  | A27 | *Aspergillus* sp. | 28.6 | 69.9±0.12 | 88.5±0.69 |
|  | A28 | *Aspergillus* sp. | 28.6 | 23.7±0.03 | - |
|  | A29 | *Penicillium* sp. | 28.6 | 93.2±0.09 | 69.3±0.95 |
|  | A30 | *Aspergillus* sp*.* | 14.6 | 83.2±0.79 | - |
| CF: Colonization frequency %  DPPH scavenging % values represents the mean ± SE, p ≤ 0.05. the positive control for DPPH scavenging (%) is ascorbic acid at 94.25 ± 0.05 | | | | | |

| **Table S2** Antimicrobial activity of fungal extracts of endophytic fungi on different pathogenic microorganisms. | | | | | | | | | | | | |
| --- | --- | --- | --- | --- | --- | --- | --- | --- | --- | --- | --- | --- |
| **Bacterial strains** | | | | | | | | | **Fungal strains** | | | |
| **Gram +Ve** | | | | | **Gram-Ve** | | | |  |  |  |  |
| **Fungal isolate** | ***Staphylococcus aureus*** | | ***Bacillus subtilis*** | | ***Escherichia coli*** | | ***Enterobacter cloacae*** | | ***Aspergillus fumigatus*** | | ***Candida albicans*** | |
|  | **Intra** | **Extra** | **Intra** | **Extra** | **Intra** | **Extra** | **Intra** | **Extra** | **Intra** | **Extra** | **Intra** | **Extra** |
| **A1** | **26.7^b^±0.31** | **30.1^a^±0.17** | **29.4^b^±0.15** | **36.13^a^±0.15** | **30.3^c^ ±0.06** | **35.15^a^ ±0.13** | **28.1^bcd^ ±0.08** | **33.17^a^ ±0.15** | **18.5^b^ ±0.04** | **19.83^a^ ±0.15** | **23.2^bc^ ±0.04** | **28.03^a^ ±0.06** |
| **A2** | 25.12^cd^±.0.11 | 26.35^bc^±0.27 | 28.95^b^±0.2 | 30.03^b^±0.11 | 29.41^e^ ±0.21 | 32.25^b^ ±0.23 | 28.97^bc^ ±0.41 | 31.17^ab^ ±0.08 | 17.68^bc^ ±0.1 | 18.32^b^ ±0.11 | 22.29^bcd^ ±0.09 | 24.01^b^ ±0.20 |
| **A3** | 24.91^d^±0.46 | 25.69^bcd^±0.26 | 26.39^c^±0.10 | 29.37^b^±0.09 | 28.74^de^ ±0.09 | 30.02^cd^ ±0.87 | 27.21^cd^ ±0.1 | 28.25^bcd^ ±0.09 | 16.98^cd^±0.46 | 17.96^bc^ ±0.15 | 21.48^b-e^ ±0.10 | 23.85^b^ ±0.29 |
| **A4** | 11.54^qrs^±0.26 | 15.20^jkl^ ±0.20 | - | 19.13^hi^±0.11 | 13.25^no^ ±0.11 | 19.97^h^ ±0..15 | - | 22.00^efg^ ±0.30 | - | 15.36^f^ ±0.09 | 6.89^wxy^ ±0.26 | 20.22^de^ ±0.18 |
| **A5** | 7.96^xy^±0.42 | 14.22^lmn^ ±0.10 | 6.05^xy^±0.03 | 11.65^o-r^ ±0.13 | - | 10.03^q-t^ ±0.55 | 8.06^wx^ ±0.04 | 13.85^n-r^ ±0.26 | 5.05^uv^ ±0.03 | 9.81^mn^ ±0.26 | 4.98^yz^ ±0.21 | 7.87^u-x^ ±0.61 |
| **A6** | - | 13.12^nop^ ±0.11 | 2.36^z^±0.11 | 9.78^r-u^ ±0.2 | - | 8.94^stu^ ±0.06 | - | 6.87^x^ ±0.23 | 7.27^qrs^ ±0.08 | 10.74^lm^ ±0.21 | 3.66^z^ ±0.11 | 9.12^r-w^ ± 0.1 |
| **A7** | 5.42^z^±0.40 | 13.77^mn^±0.28 | - | 10.96^p-s^ ±0.22 | 6.22^w^ ±0.19 | 16.33^ijk^ ±0.34 | - | 12.74^o-t^ ±0.28 | 4.36^v^ ±0.1 | 13.12^gh^ ±0.27 | 7.36^v-y^ ±.011 | 11.89^m-q^ ±0.26 |
| **A8** | 12.15^o-r^ ±0.11 | 19.37^f^±0.24 | 15.82^lm^±0.09 | 20.41^fgh^ ±0.19 | 14.11^mn^ ±0.07 | 21.92^g^ ±0.56 | 13.71^n-s^ ±0.26 | 19.55^f-j^ ±0.38 | - | - | 11.16^m-s^ ±0.09 | 17.35^f-i^ ±0.22 |
| **A9** | - | 22.33^e^±0.02 | 7.37 ^VWX^ ±0.11 | 16.71^jkl^±0.21 | 15.15^klm^ ±0.1 | 26.24^f^ ±0.18 | 17.22^i-n^ ±0.11 | 25.19^de^ ±0.82 | 9.61^no^ ±0.11 | 10.98^kl^ ±0.04 | - | - |
| **A10** | 16.7 ^ghi^ ±0.30 | 20.75^f^±0.03 | - | 22.23^ef^±0.2 | - | 9.66^r-u^ ±0.57 | - | - | - | 15.6^ef^ ±0.30 | - | 19^efg^ ±0.01 |
| **A11** | - | 10.93^r-u^ ±0.12 | 17.36 ^IJKL^ ±0.19 | 24.10^de^±0.10 | 8.7^tuv^ ±0.04 | 12.93^no^ ±0.40 | - | 26.03^cd^ ±0.15 | 9.88^mn^ ±0.26 | 11.83^ijk^ ±0.21 | 13.77^j-n^ ±0.09 | 20.60^cde^ ±0.53 |
| **A12** | 14.21^lmn^±0.10 | 20.6^f^±0.27 | - | 21.98^f^±0.11 | - | 14.33^lmn^ ±0.0=21 | 13^o-t^ ±0.09 | 19.14^f-k^ ±0.11 | - | - | 9.35^q-w^ ±0.21 | 11.85^m-r^ ±0.23 |
| **A13** | - | 9.86^uf^±0.11 | 7.21 ^VWX^ ±0.11 | 12.54^nop^ ±0.19 | 11.23^pq^ ±017 | 17.46^i^ ±0.09 | 12.56^o-u^ ±0.08 | 18.2^h-l^ ±0.04 | 6.25 ^t^ ±0.18 | 10.96^kl^ ±0.1 | 8.99^s-w^ ±0.33 | 13.25^l-o^ ±0.11 |
| **A14** | 13.25^nop^±0.12 | 15.74^ijk^ ±0.31 | - | 11.58^o-r^ ±0.11 | 14.37^lmn^ ±0.21 | 19.11^h^ ±0.1 | 12.42^p-u^ ±0.21 | 20.26^f-i^ ±00.12 | 5.99^tu^ ±0.51 | 9.25^no^ ±0.11 | - | - |
| **A15** | 11.25^q-t^±0.20 | 17.22^gh^ ±0.16 | 16.35^kl^±0.06 | 19.89^gh^ ±0.32 | - | - | - | 21.25^fgh^ ±0.11 | 11.37^jkl^±0.17 | 16.52^de^ ±0.11 | 13.58^k-o^ ±0.10 | 19.28^ef^ ±0.17 |
| **A16** | - | - | 10.25^q-t^ ±0.12 | 13.95^mn^±0.35 | - | 22.13^g^ ±0.11 | 15.31^l-p^ ±0.07 | 17.99^h-m^ ±0.46 | - | 6.32^st^ ±0.08 | 9.15^q-w^ ±0.08 | 13.24^l-o^ ±0.08 |
| **A17** | 9.89^tuv^ ±0.08 | 14.88^klm^ ±0.17 | - | - | 10.15^q-t^ ±0.12 | 15.74^jkl^ ±0.31 | 11.22^q-w^ ±0.1 | 13.58^n-t^ ±0.14 | 8.65 ^op^ ±0.27 | 12.18^hij^ ±0.13 | 7.25^v-y^ ±0.19 | 18.77^e-h^ ±0.21 |
| **A18** | 8.44^wxy^ ±0.21 | 22.97e±0.15 | 12.38^nop^ ±0.11 | 21.27fg±0.25 | 9.55^r-u^ ±0.22 | 16.93^ij^ ±0.21 | 13.25^o-t^ ±0.11 | 22.07^ef^ ±0.31 | - | 8.22^pq^ ±0.11 | - | 16.19^h-k^ ±0.17 |
| **A19** | - | - | 4.56 y±0.09 | - | - | - | 10.12^s-x^ ±0.1 | 14.32^m-q^ ±0.09 | - | 15.31^f^ ±0.21 | - | - |
| **A20** | 9^vwx^ ±0.06 | 13.01^nop^ ±0.02 | 12.14^n-q^ ±0.09 | 19.24^hi^±0.21 | 14.32^lmn^ ±0.20 | 20.34^h^ ±0.22 | - | 10.01^t-x^ ±0.02 | 9.41^no^ ±0.21 | 13.24^g^ ±0.1 | 10.34^p-u^ ±0.21 | 14.85^i-l^ ±0.30 |
| **A21** | 14.12^lmn^±0.13 | 17.76^g^ ±0.19 | 11.43^o-r^ ±0.09 | 18.58^hij^ ±0.09 | - | 8.36^uv^ ±29 | 9.01^u-x^ ±0.03 | 11.26^q-w^ ±0.09 | - | 9.12^nop^ ±0.09 | - | 15.16^i-l^ ±0.11 |
| **A22** | 7.36^y^±0.22 | 14.19^lmn^ ±0.12 | - | 5.65^xy^ ±0.21 | 12.01^op^ ±0.02 | 14.97^klm^ ±0.11 | 15.66^k-p^ ±0.22 | 17.74^h-m^ ±0.09 | - | 14.65^f^ ±0.11 | - | - |
| **A23** | - | - | 13^no^±0.01 | 17.86^ijk^ ±0.09 | - | 16.03^ijk^ ±0.1 | - | - | 6.68^rst^ ±0.26 | 11.32^jkl^ ±0.20 | - | 13.89^j-m^ ±0.10 |
| **A24** | 14.01^lmn^±1.01 | 16.44^g-j^ ±0.08 | - | 15.6^lm^ ±0.1 | 17.16^ij^ ±0.09 | 19.52^h^ ±0.26 | - | 16.22^j-o^ ±0.22 | - | - | 6.9^wxy^ ±0.35 | 11.01^n-t^ ±0.11 |
| **A25** | - | 9.77^uvw^ ±0.20 | 9.01^tuv^ ±0.04 | 11.35^o-r^ ±0.02 | - | 12.11^op^ ±0.09 | 10.45^r-x^ ±0.18 | 13.21^o-t^ ±0.1 | 7.55^qr^ ±0.06 | 10.69^lm^ ±0.12 | 9.85^p-v^ ±0.1 | 12.55^l-p^ ±0.21 |
| **A26** | - | - | 8.08^uvw^ ±0.03 | 10.25^q-t^ ±0.19 | 11.36^pq^ ±0.23 | 15.78^jkl^ ±0.11 | - | 18.33^g-l^ ±0.19 | 5.99^tu^ ±0.33 | 9.35^no^ ±0.23 | - | 10.99^o-t^ ±0.40 |
| **A27** | 10.5^st^±0.28 | 12.36^opq^ ±0.28 | - | 9.36^stu^ ±0.11 | 10.98^pqr^ ±0.04 | 13.65^mn^ ±0.3 | 11.35^q-w^ ±0.1 | 14.79^l-q^ ±0.19 | - | 7.33^qr^ ±0.09 | 7.64^u-y^ ±0.20 | 16.4^g-j^ ±0.15 |
| **A28** | - | 11.95^pqr^ ±0.06 | 11.43^o-r^ ±0.2 | 16.87^jkl^ ±0.09 | - | 7.35^vw^ ±0.07 | 8.55^wx^ ±0.09 | 10.36^r-x^ ±0.23 | 8.64^op^ ±0.21 | 12.4^ghi^ ±0.37 | - | 8.25^t-x^ ±0.1 |
| **A29** | 16.35 ^hij^ ±0.12 | 19.61^f^±0.43 | - | 17.05^jkl^ ±0.04 | - | 16.84^ij^ ±0.21 | - | 15.33^l-p^ ±0.21 | - | 15.49 ^f^ ±0.09 | 6.19 ^xyz^ ±0.9 | 11.16^m-s^ ±0.09 |
| **A30** | 11.54^qrs^±0.36 | 13.5^mno^ ±0.19 | 7.02^wx^ ±0.01 | 9.93^r-u^ ±0.26 | 8.66^tuv^ ±0.24 | 10.23^qrs^ ±0.1 | 8.69 ^vwx^ ±0.28 | 12.25^p-v^ ±0.13 | 4.25^v^ ±0.1 | 8.95 ^nop^ ±0.11 | 5.97 ^xyz^ ±0.38 | 9.26^q-w^ ±0.22 |
| **Control** | 27.03 b± 0.06 | | 26.02^cd^ ± 0.03 | | 29.90^cd^ ± 0.01 | | 27.03^cd^ ± 0.06 | | 17.01^cd^ ± 0.02 | | 20.03^def^ ± 0.04 | |
| Means in each two columns with similar letters are not significantly different at the 0.05 level of significance, according to Duncan’s multiple range test.  Extra: refers to the extracellular extract of fungus  Intra: refers to the intracellular extract of fungus  The test was done using the diffusion agar technique, well diameter: 6.0 mm (100 µl was tested), Data represented as mean ± standard deviation (SD).Positive control for fungi: Ketoconazole 100µg/ml. Positive control for +ve and –ve bacteria: Gentamycin 4µg/ml. | | | | | | | | | | | | |

| **Table S3** Collection Table of final concentrated fraction obtained by flash chromatography. | | | | | | | | | | | | |
| --- | --- | --- | --- | --- | --- | --- | --- | --- | --- | --- | --- | --- |
| **Tube** | **Peak** | **Rack** | **Pos.** | **R Set** | **Coll.** | **Volume** | **Surface** | **%Surface** | **Start Time** | **End Time** | **Begin** | **End** |
| 001 | 001 | 1 | 1 | 1 | 1 | 20.0 | -0.0 | -0.0 % | 00:00:01 | 00:01:21 | 0.01 | 0.63 |
| 002 | 002 | 1 | 2 | 1 | 1 | 20.0 | -0.0 | -0.0 % | 00:01:21 | 00:02:41 | 0.63 | 1.26 |
| 003/006 | 003 | 1 | 3/6 | 1 | 1 | 79.8 | -0.0 | -0.0 % | 00:02:41 | 00:08:00 | 1.26 | 3.75 |
| 007/013 | 004 | 1 | 7/13 | 1 | 1 | 125.0 | 0.0 | 0.0 % | 00:08:00 | 00:16:20 | 3.75 | 7.66 |
| 014 | 005 | 1 | 14 | 1 | 1 | 20.0 | 0.6 | 0.9 % | 00:16:20 | 00:17:40 | 7.66 | 8.28 |
| 015/016 | 006 | 1 | 15/16 | 1 | 1 | 40.0 | 0.6 | 0.9 % | 00:17:40 | 00:20:20 | 8.28 | 9.53 |
| 017 | 007 | 1 | 17 | 1 | 1 | 15.0 | 0.4 | 0.6 % | 00:20:20 | 00:21:20 | 9.53 | 10.00 |
| 018 | 008 | 1 | 18 | 1 | 1 | 20.0 | 0.4 | 0.6 % | 00:21:20 | 00:22:40 | 10.00 | 10.63 |
| 019/020 | 009 | 1 | 19/20 | 1 | 1 | 29.0 | 0.4 | 0.6 % | 00:22:40 | 00:24:36 | 10.63 | 11.53 |
| 021 | 010 | 1 | 21 | 1 | 1 | 4.0 | 0.1 | 0.2 % | 00:24:36 | 00:24:52 | 11.53 | 11.66 |
| 022 | 011 | 1 | 22 | 1 | 1 | 0.8 | 0.0 | 0.0 % | 00:24:52 | 00:24:55 | 11.66 | 11.68 |
| 023/025 | 012 | 1 | 23/25 | 1 | 1 | 32.0 | 1.7 | 2.6 % | 00:24:55 | 00:27:03 | 11.68 | 12.68 |
| 026 | 013 | 1 | 26 | 1 | 1 | 0.5 | 0.0 | 0.0 % | 00:27:03 | 00:27:05 | 12.68 | 12.70 |
| 027 | 014 | 1 | 27 | 1 | 1 | 9.5 | 0.2 | 0.3 % | 00:27:05 | 00:27:43 | 12.70 | 12.99 |
| 028/029 | 015 | 1 | 28/29 | 1 | 1 | 38.0 | 0.6 | 0.9 % | 00:27:43 | 00:30:15 | 12.99 | 14.18 |
| 030 | 016 | 1 | 30 | 1 | 1 | 3.5 | 0.1 | 0.2 % | 00:30:15 | 00:30:29 | 14.18 | 14.29 |
| 031 | 017 | 1 | 31 | 1 | 1 | 15.0 | 0.4 | 0.6 % | 00:30:29 | 00:31:29 | 14.29 | 14.76 |
| 032/033 | 018 | 1 | 32/33 | 1 | 1 | 35.0 | 0.8 | 1.2 % | 00:31:29 | 00:33:49 | 14.76 | 15.85 |
| 034 | 019 | 1 | 34 | 1 | 1 | 17.0 | 0.3 | 0.5 % | 00:33:49 | 00:34:57 | 15.85 | 16.38 |
| 035/042 | 020 | 1 | 35/42 | 1 | 1 | 155.0 | 2.7 | 4.1 % | 00:34:57 | 00:45:17 | 16.38 | 21.23 |
| 043/044 | 021 | 1 | 43/44 | 1 | 1 | 30.0 | 0.7 | 1.1 % | 00:45:17 | 00:47:17 | 21.23 | 22.16 |
| 045/058 | 021 | 2 | 1/14 | 1 | 1 | 205.5 | 7.1 | 10.8 % | 00:47:17 | 01:00:59 | 22.16 | 28.59 |
| 043/058 | 021 | - | - | - | - | 235.5 | 7.8 | 11.9 % | 00:45:17 | 01:00:59 | 21.23 | 28.59 |
| 059 | 022 | 2 | 15 | 1 | 1 | 15.0 | 0.5 | 0.8 % | 01:00:59 | 01:01:59 | 28.59 | 29.05 |
| 060/061 | 023 | 2 | 16/17 | 1 | 1 | 23.5 | 0.9 | 1.4 % | 01:01:59 | 01:03:33 | 29.05 | 29.79 |
| 062 | 024 | 2 | 18 | 1 | 1 | 1.0 | 0.0 | 0.0 % | 01:03:33 | 01:03:37 | 29.79 | 29.82 |
| 063/064 | 025 | 2 | 19/20 | 1 | 1 | 15.3 | 0.7 | 1.1 % | 01:03:37 | 01:04:38 | 29.82 | 30.30 |
| 065 | 026 | 2 | 21 | 1 | 1 | 0.3 | 0.0 | 0.0 % | 01:04:38 | 01:04:39 | 30.30 | 30.30 |
| 066/088 | 027 | 2 | 22/44 | 1 | 1 | 345.0 | 20.4 | 31.1 % | 01:04:39 | 01:27:39 | 30.30 | 41.09 |
| 089/104 | 027 | 3 | 1/16 | 1 | 1 | 240.0 | 16.2 | 24.7 % | 01:27:39 | 01:43:39 | 41.09 | 48.59 |
| 066/104 | 027 | - | - | - | - | 585.0 | 36.6 | 55.9 % | 01:04:39 | 01:43:39 | 30.30 | 48.59 |
| 105 | 028 | 3 | 17 | 1 | 1 | 3.5 | 0.1 | 0.2 % | 01:43:39 | 01:43:53 | 48.59 | 48.70 |
| 106 | 029 | 3 | 18 | 1 | 1 | 15.0 | 0.6 | 0.9 % | 01:43:53 | 01:44:53 | 48.70 | 49.16 |
| 107/113 | 030 | 3 | 19/25 | 1 | 1 | 103.8 | 3.5 | 5.3 % | 01:44:53 | 01:51:48 | 49.16 | 52.41 |
| 114 | 031 | 3 | 26 | 1 | 1 | 6.5 | 0.2 | 0.3 % | 01:51:48 | 01:52:14 | 52.41 | 52.61 |
| 115 | 032 | 3 | 27 | 1 | 1 | 1.5 | 0.0 | 0.0 % | 01:52:14 | 01:52:20 | 52.61 | 52.66 |
| 116 | 033 | 3 | 28 | 1 | 1 | 1.5 | 0.0 | 0.0 % | 01:52:20 | 01:52:26 | 52.66 | 52.70 |
| 117 | 034 | 3 | 29 | 1 | 1 | 4.0 | 0.1 | 0.2 % | 01:52:26 | 01:52:42 | 52.70 | 52.83 |
| 118 | 035 | 3 | 30 | 1 | 1 | 1.8 | 0.1 | 0.2 % | 01:52:42 | 01:52:49 | 52.83 | 52.88 |
| 119 | 036 | 3 | 31 | 1 | 1 | 0.8 | 0.0 | 0.0 % | 01:52:49 | 01:52:52 | 52.88 | 52.91 |
| 120 | 037 | 3 | 32 | 1 | 1 | 0.8 | 0.0 | 0.0 % | 01:52:52 | 01:52:55 | 52.91 | 52.93 |
| 121 | 038 | 3 | 33 | 1 | 1 | 0.5 | 0.0 | 0.0 % | 01:52:55 | 01:52:57 | 52.93 | 52.95 |
| 122 | 039 | 3 | 34 | 1 | 1 | 2.8 | 0.1 | 0.2 % | 01:52:57 | 01:53:08 | 52.95 | 53.03 |
| 123 | 040 | 3 | 35 | 1 | 1 | 0.8 | 0.0 | 0.0 % | 01:53:08 | 01:53:11 | 53.03 | 53.05 |
| 124 | 041 | 3 | 36 | 1 | 1 | 8.0 | 0.3 | 0.5 % | 01:53:11 | 01:53:43 | 53.05 | 53.30 |
| 125 | 042 | 3 | 37 | 1 | 1 | 2.3 | 0.1 | 0.2 % | 01:53:43 | 01:53:52 | 53.30 | 53.38 |
| 126 | 043 | 3 | 38 | 1 | 1 | 3.0 | 0.1 | 0.2 % | 01:53:52 | 01:54:04 | 53.38 | 53.47 |
| 127 | 044 | 3 | 39 | 1 | 1 | 14.0 | 0.5 | 0.8 % | 01:54:04 | 01:55:00 | 53.47 | 53.91 |
| 128 | 045 | 3 | 40 | 1 | 1 | 15.0 | 0.5 | 0.8 % | 01:55:00 | 01:56:00 | 53.91 | 54.38 |
| 129/130 | 046 | 3 | 41/42 | 1 | 1 | 24.5 | 1.0 | 1.5 % | 01:56:00 | 01:57:38 | 54.38 | 55.14 |
| 131 | 047 | 3 | 43 | 1 | 1 | 0.8 | 0.0 | 0.0 % | 01:57:38 | 01:57:41 | 55.14 | 55.16 |
| 132 | 048 | 3 | 44 | 1 | 1 | 2.8 | 0.1 | 0.2 % | 01:57:41 | 01:57:52 | 55.16 | 55.25 |
| 133 | 049 | 4 | 1 | 1 | 1 | 7.5 | 0.3 | 0.5 % | 01:57:52 | 01:58:22 | 55.25 | 55.48 |
| 134 | 050 | 4 | 2 | 1 | 1 | 1.3 | 0.1 | 0.2 % | 01:58:22 | 01:58:27 | 55.48 | 55.52 |
| 135 | 051 | 4 | 3 | 1 | 1 | 0.5 | 0.0 | 0.0 % | 01:58:27 | 01:58:29 | 55.52 | 55.54 |
| 136 | 052 | 4 | 4 | 1 | 1 | 1.8 | 0.1 | 0.2 % | 01:58:29 | 01:58:36 | 55.54 | 55.59 |
| 137 | 053 | 4 | 5 | 1 | 1 | 1.0 | 0.0 | 0.0 % | 01:58:36 | 01:58:40 | 55.59 | 55.63 |
| 138/139 | 054 | 4 | 6/7 | 1 | 1 | 22.3 | 0.9 | 1.4 % | 01:58:40 | 02:00:09 | 55.63 | 56.32 |
| 140 | 055 | 4 | 8 | 1 | 1 | 2.8 | 0.1 | 0.2 % | 02:00:09 | 02:00:20 | 56.32 | 56.41 |
| 141 | 056 | 4 | 9 | 1 | 1 | 15.0 | 0.5 | 0.8 % | 02:00:20 | 02:01:20 | 56.41 | 56.88 |
| 142 | 057 | 4 | 10 | 1 | 1 | 12.8 | 0.4 | 0.6 % | 02:01:20 | 02:02:11 | 56.88 | 57.27 |

| **Table S4** Elution steps of sub-fractions |
| --- |
| 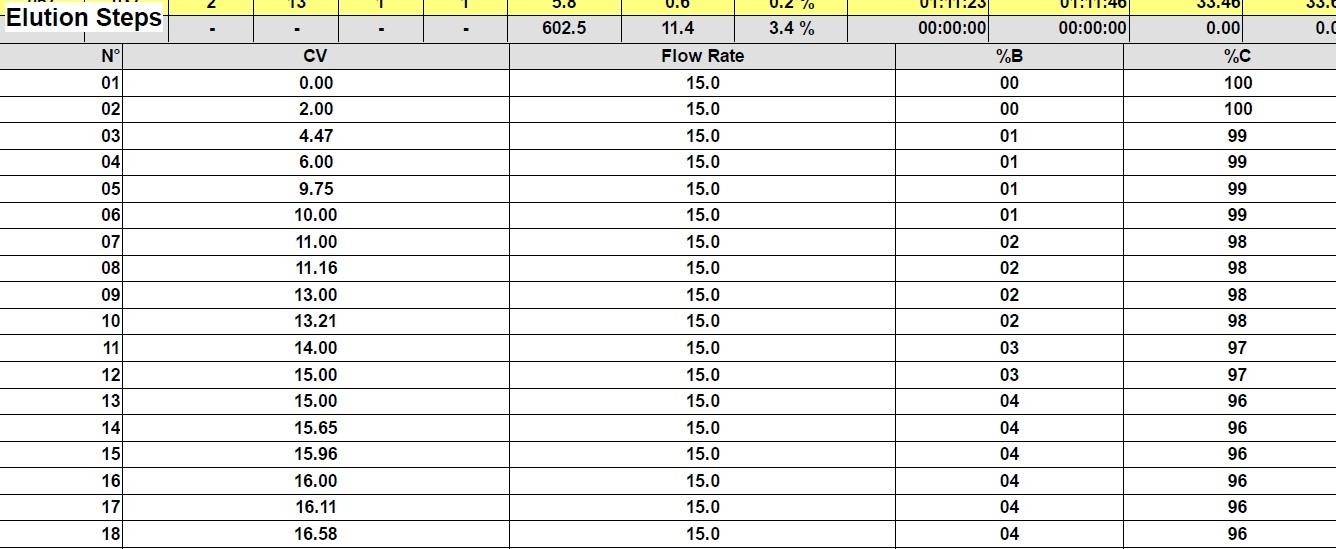 **Number**  **of test tube**  **Solvent system percentage at which the analyte eluted.**  **Number of CVs (column volumes) required to elute an analyte from a column** |

| **Table S5** DPPH scavenging activity of HDOCOX with IC_50_ | | |
| --- | --- | --- |
| **Concentration (µg/mL)** | **DPPH scavenging activity %** | |
|  | **Ascorbic acid** | **HDOCOX** |
| 0 | 0.00^i^ | 0.00^i^ |
| 5 | 13.25 ± 0.02^h^ | 8.2 ± 0.062^h^ |
| 10 | 21.36 ± 0.026^g^ | 11.4 ± 0.02^g^ |
| 20 | 52.65 ± 0.15^f^ | 19.4 ± 0.13^f^ |
| 40 | 66.56 ± 0.082^e^ | 32.2 ± 0.2^e^ |
| 80 | 73.62 ± 0.067^d^ | 42.5 ± 0.55^d^ |
| 160 | 80.75 ± 0.36^c^ | 53.7 ± 0.79^c^ |
| 320 | 89.34 ± 0.049^b^ | 69.3 ± 0.58^b^ |
| 640 | 94.25 ± 0.05^a^ | 94.1 ± 079^a^ |
| Means ± SE with different letters in the same columns are significantly different according to Duncan’s multiple range test at p ≤ 0.05. | | |

| **Table S6** MICs of MnNPs, HDOCOX, and MnNCs against *E.coli* and *C. albicans* | | | |
| --- | --- | --- | --- |
| **Antimicrobial agents Conc. (µg/mL)** | | **Diameter of inhibition zone (mm)** | |
|  |  | ***E.coli*** | ***C. albicans*** |
| **MnNPs** | **5** | 0.000 ^j^±0.00 | 0.000 ^k^±0.00 |
|  | **10** | 0.000 ^j^±0.00 | 0.000^k^±0.00 |
|  | **20** | 16.13 ^i^±0.13 | 15.50 ^j^±0.40 |
|  | **30** | 18.16 ^g^±0.08 | 16.60 ^i^±0.35 |
|  | **40** | 22.83 ^f^±0.58 | 20.26 ^h^±0.03 |
|  | **50** | 26.96 ^e^±0.89 | 23.26 ^fg^±0.57 |
|  | **70** | 31.70 ^d^±0.65 | 27.10^d^±0.10 |
|  | **100** | 35.83^c^±0.44 | 30.63 ^c^±0.44 |
| **HDOCOX** | **5** | 0.000 ^j^±0.00 | 0.000 ^k^±0.00 |
|  | **10** | 0.000 ^j^±0.00 | 0.000 ^k^±0.00 |
|  | **20** | 16.50 ^hi^±0.25 | 15.50 ^j^±0.36 |
|  | **30** | 17.76 ^gh^±0.39 | 17.26 ^i^±0.57 |
|  | **40** | 22.20^f^±0.66 | 19.26 ^h^±0.60 |
|  | **50** | 26.16 ^e^±0.08 | 24.26 ^f^±0.54 |
|  | **70** | 31.03 ^d^±1.31 | 25.86 ^e^±0.43 |
|  | **100** | 35.30 ^c^±0.17 | 27.50 ^d^±0.05 |
| **MnNCs** | **5** | 0.000 ^j^±0.00 | 0.000 ^k^±0.00 |
|  | **10** | 26.20 ^e^±0.26 | 23.10 ^g^±0.10 |
|  | **20** | 32.10 ^d^±0.66 | 23.56^fg^±0.03 |
|  | **30** | 38.16 ^b^±1.08 | 30.56 ^c^±0.08 |
|  | **40** | 38.73^b^±0.37 | 31.60 ^c^±0.65 |
|  | **50** | 37.86^b^±0.98 | 31.26 ^c^±0.14 |
|  | **70** | 39.13 ^b^±0.86 | 33.40 ^b^±0.94 |
|  | **100** | 42.63 ^a^±0.26 | 36.06 ^a^±0.29 |
| Means ±SE with different letters in the same columns are significantly different according to Duncan’s multiple range test at p ≤ 0.05.  Highlighted cells refers to the MICs of each antimicrobial agent. The test was done using the diffusion agar technique, well diameter: 6.0 mm (100 µl was tested), Data represented as mean ± standard deviation (SD).Positive control for fungi: Ketoconazole 100µg/ml. Positive control for +ve and –ve bacteria: Gentamycin 4µg/ml. | | | |

**Table S7** Detected cytotoxicity at different concentrations of MnNps, HDOCOX and MnNCs against HepG-2 cell line.

|  | | | |
| --- | --- | --- | --- |
| **Compounds** | **Conc. (µg/ml)** | **Viability (%)** | **Inhibitory (%)** |
| **MnNPS** | 500 | 5.43^a^ | 94.57^i^ |
|  | 250 | 13.92^b^ | 86.08^h^ |
|  | 125 | 28.87^c^ | 71.13^g^ |
|  | 62.5 | 43.19^d^ | 56.81^f^ |
|  | 31.25 | 75.24^e^ | 24.76^e^ |
|  | 15.6 | 88.72^f^ | 11.28^d^ |
|  | 7.8 | 94.13^g^ | 5.87^c^ |
|  | 3.9 | 98.76^h^ | 1.24^b^ |
|  | 0 | 100^i^ | 0^a^ |
| **HDOCOX** | 500 | 3.48^a^ | 96.52^i^ |
|  | 250 | 6.79^b^ | 93.21^h^ |
|  | 125 | 15.22^c^ | 84.78^g^ |
|  | 62.5 | 28.97^d^ | 71.03^f^ |
|  | 31.25 | 42.81^e^ | 57.19^e^ |
|  | 15.6 | 69.40^f^ | 30.6^d^ |
|  | 7.8 | 88.06^g^ | 11.94^c^ |
|  | 3.9 | 97.14^h^ | 2.86^b^ |
|  | 0 | 100^i^ | 0^a^ |
| **MnNCs** | 500 | 1.27^a^ | 98.73^i^ |
|  | 250 | 3.49^b^ | 96.51^h^ |
|  | 125 | 8.64^c^ | 91.36^g^ |
|  | 62.5 | 13.70^d^ | 86.3^f^ |
|  | 31.25 | 20.89^e^ | 79.11^e^ |
|  | 15.6 | 33.46^f^ | 66.54^d^ |
|  | 7.8 | 48.12^g^ | 51.88^c^ |
|  | 3.9 | 67.98^h^ | 32.02^b^ |
|  | 0 | 100^i^ | 0^a^ |
| Means in each column with similar letters are not significantly different at the 0.05 level of significance, according to Duncan’s multiple range test. Positive control for Inhibitory activity against Hepatocellular carcinoma cells was detected under these experimental conditions using Vinblastine Sulfate | | | |

**Supplementary Fig. 1** Endophytic fungi isolates, their antioxidant potential of their intracellular and extracellular extracts (50 µg/mL).


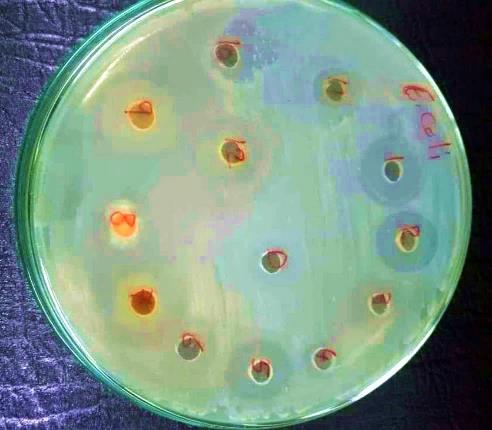

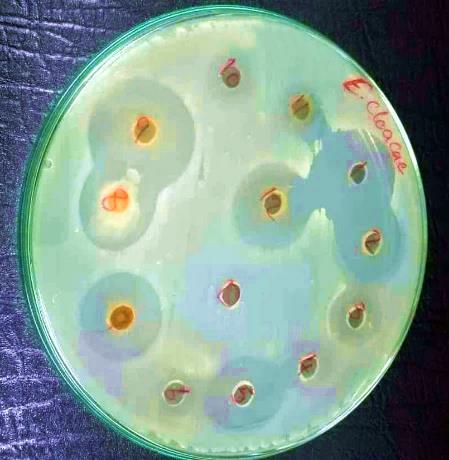


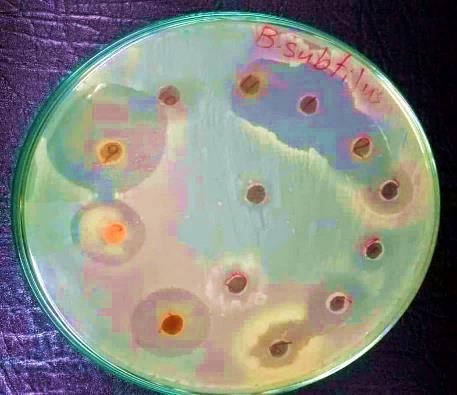

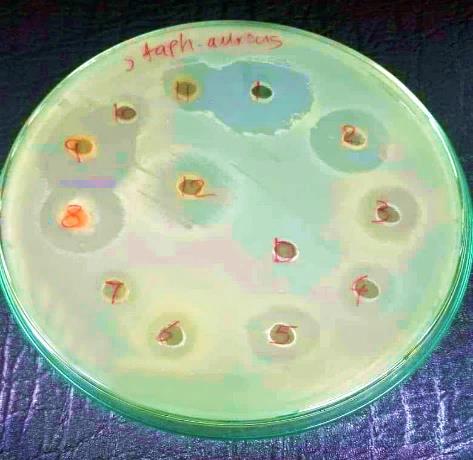


**Supplementary Fig. 2 Antibacterial effect of most active extracellular extracts of fungal isolates codes A1, A2, A3, A4, A 5, A7, A8, A10, A12, A14, A15, A17 and A18 on *E. coli*, *E. cloacae, B. subtilis* and *staph. aureus.***


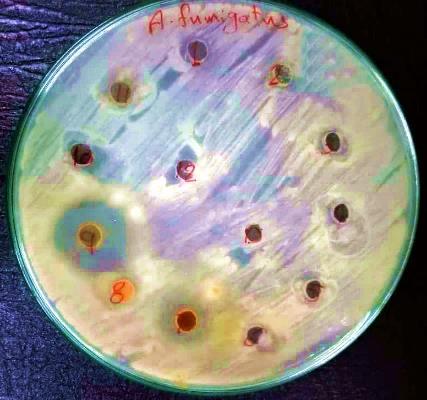

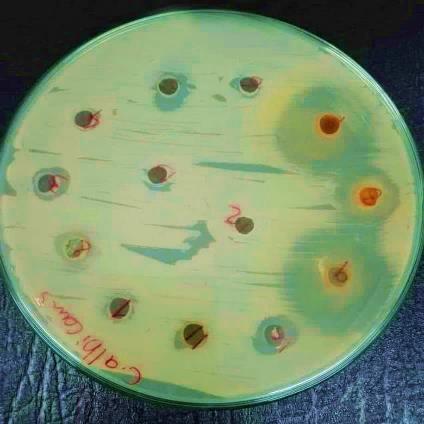


**Supplementary Fig. 3 Antifungal effect of most active extracellular extracts of fungal isolates codes A1, A2, A3, A4, A8, A10, A11, A15, A17, A18 and A27** **on** ***A. fumigatus and C. albicans***


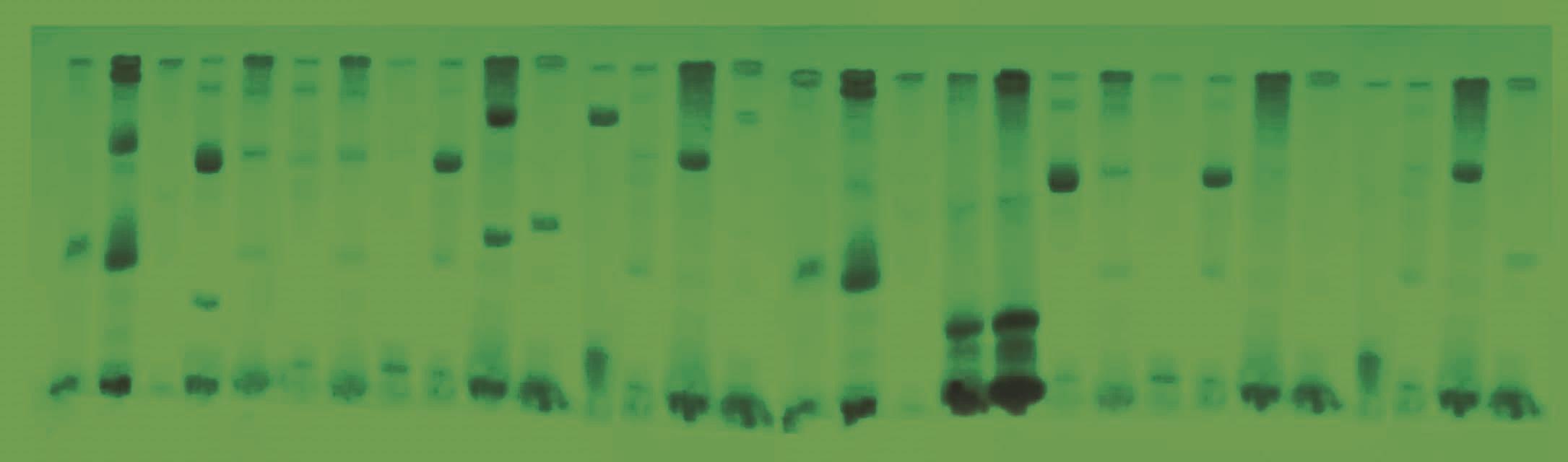


**Supplementary Fig.4.TLC chromatogram of 30 fungal secondary metabolites extracts.**

GAAGGATCATTACCGAGTGCGGGTCTTTATGGCCCAACCTCCCACCCGTGACTATTGTACCTTGTTGCTTCGGCGGGCCCGCCAGCGTTGCTGGCCGCCGGGGGGCGACTCGCCCCCGGGCCCGTGCCCGCCGGAGACCCCAACATGAACCCTGTTCTGAAAGCTTGCAGTCTGAGTGTGATTCTTTGCAATCAGTTAAAACTTTCAACAATGGATCTCTTGGTTCCGGCATCGATGAAGAACGCAGCGAAATGCGATAACTAATGTGAATTGCAGAATTCAGTGAATCATCGAGTCTTTGAACGCACATTGCGCCCCCTGGTATTCCGGGGGGCATGCCTGTCCGAGCGTCATTGCTGCCCTCAAGCCCGGCTTGTGTGTTGGGCCCTCGTCCCCCGGCTCCCGGGGGACGGGCCCGAAAGGCAGCGGCGGCACCGCGTCCGGTCCTCGAGCGTATGGGGCTTCGTCTTCCGCTCCGTAGGCCCGGCCGGCGCCCGCCGACGCATTTATTTGCAACTTGTTTTTTTCCAGGTTGACCTCGGATCAGGTAGGGATACCCGCTGAACTTAAGCATATGCG

**Supplementary Fig.5**ITS sequences nucleotides region of rDNA of the fungal sample isolated in the present study (*Aspergillus terreus* AUMC15810 ).

**
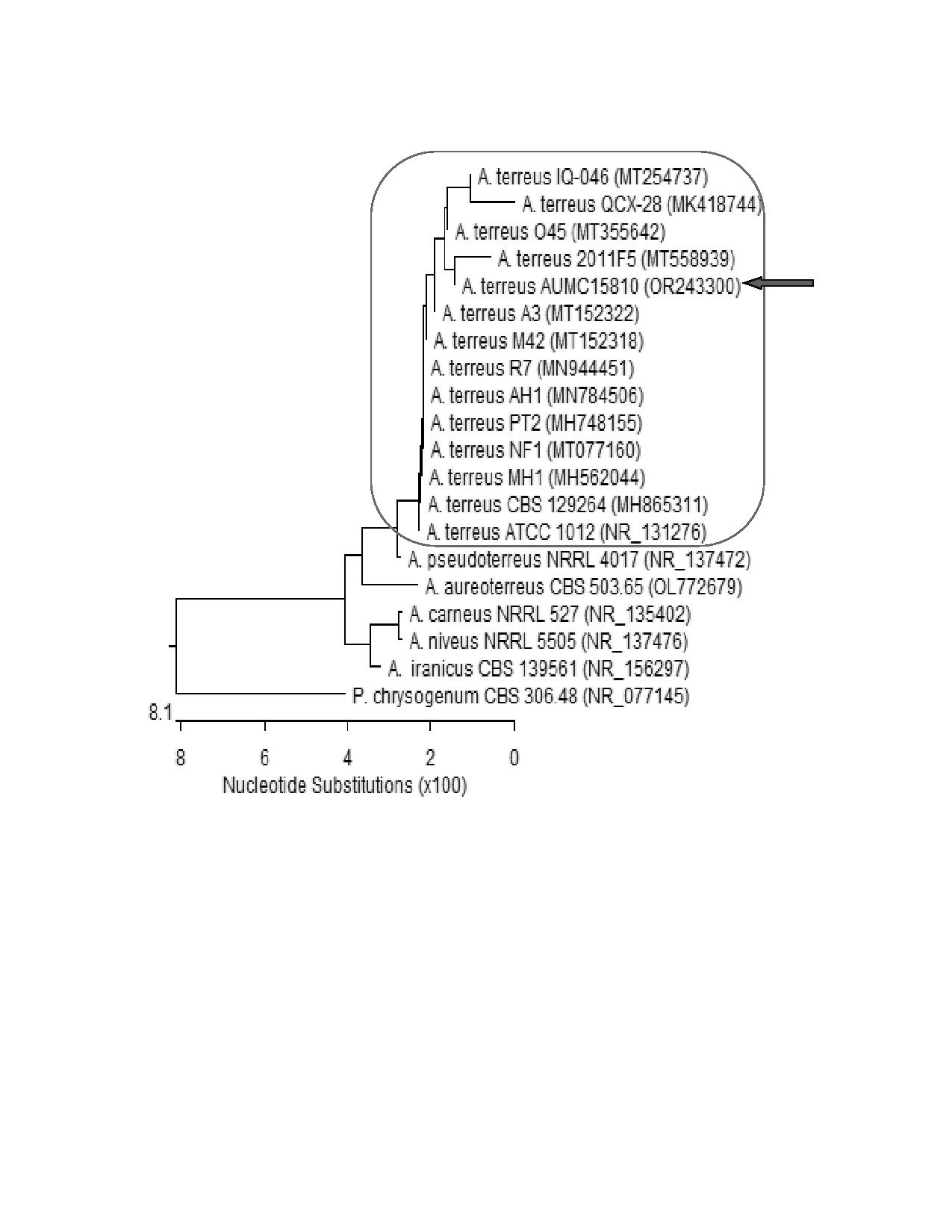
**

**Supplementary Fig 6** Phylogenetic tree based on ITS sequences of rDNA of the fungal sample isolated in the present study (*Aspergillus terreus* AUMC15810 with accession no.OR243300, arrowed) aligned with closely related strains accessed from the GenBank. This strain showed 100% identity and 100% coverage with several strains of the same species including the type material *A. terreus* ATCC1012 with GenBank accession no NR_131276. *Penicillium chrysogenum* represents an outgroup strain. A = Aspergillus, P = Penicillium


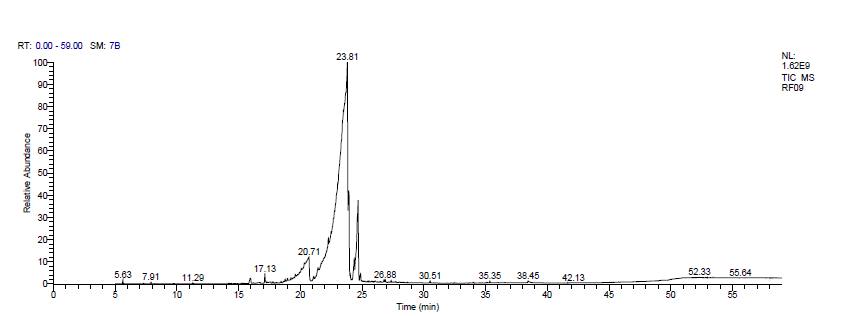


**Supplementary Fig. 7** GC-MS chromatogram of secondary metabolites of *A.terreus*.


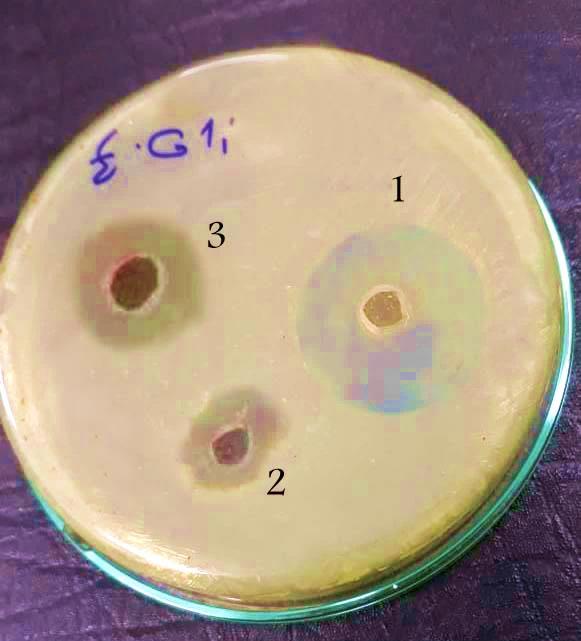

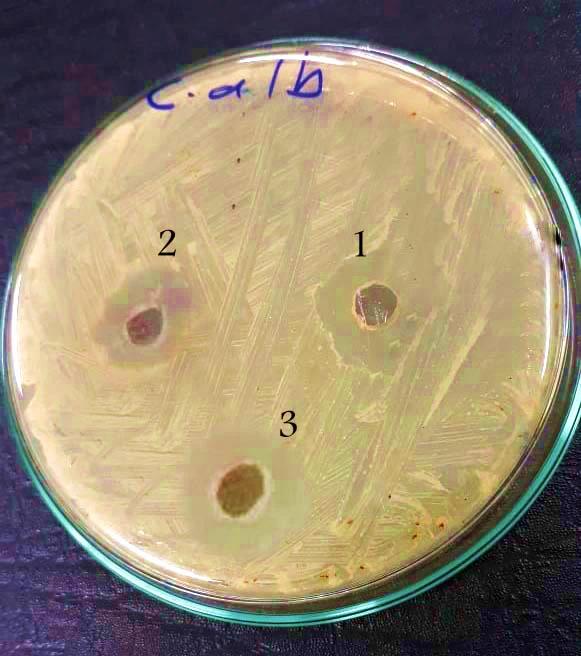


**Supplementary Fig .8 Antimicrobial effect of (1): MnNCs, 2): MnNPs, 3): HDOCOX on *E. coli* and *C. albicans.***


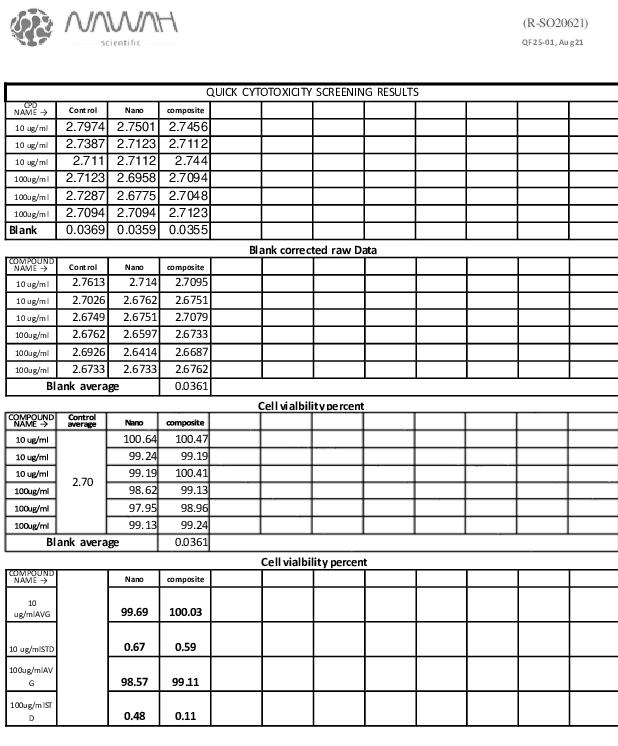


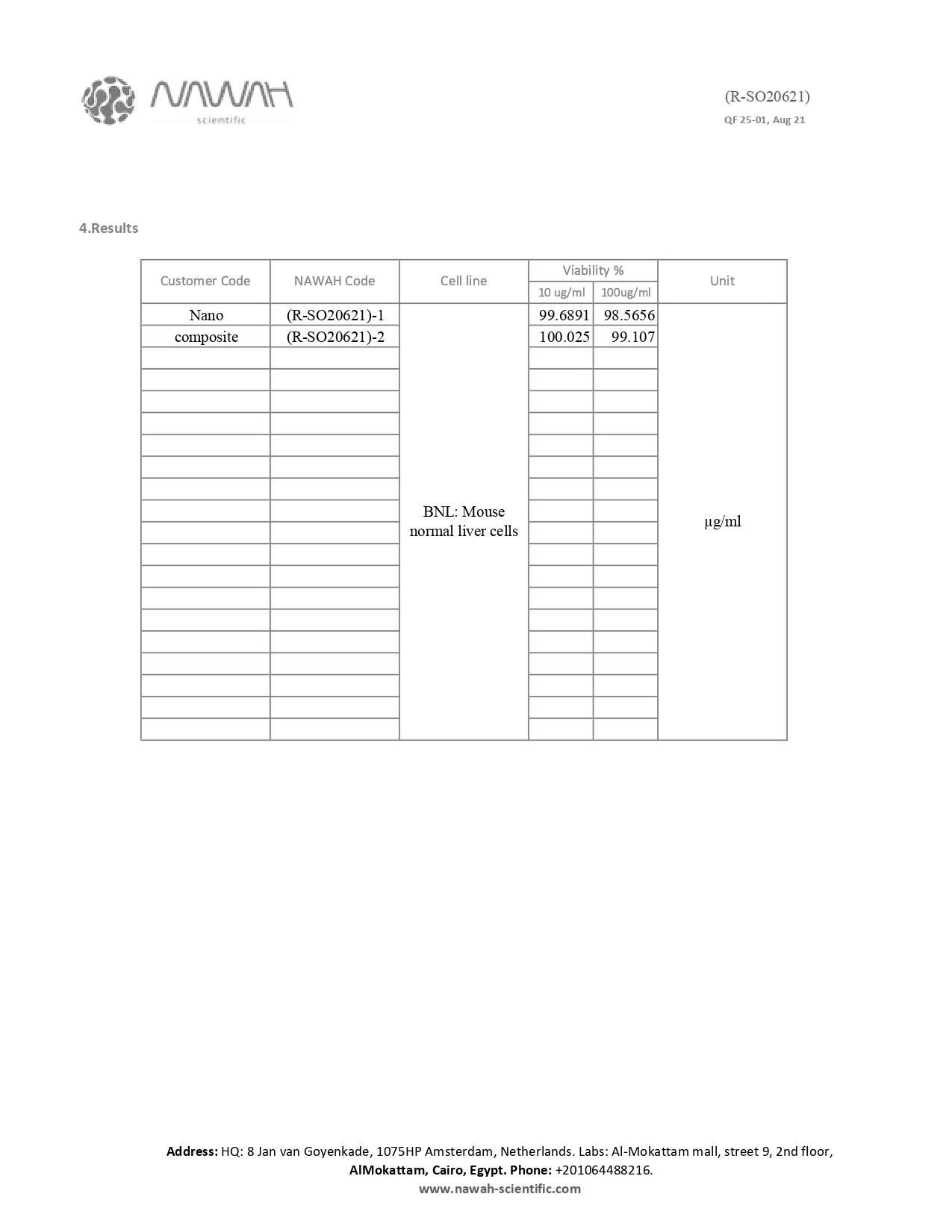


**Supplementary Fig .9 Cytotoxicity assay of MnNPS and MnNCs on normal cell line (Mouse normal liver cells) as Preliminary test for determination the cell viability at**
